# Cytotoxic Activity and Molecular Docking Studies of ONNO Type Salophen Schiff Base Metal Complexes

**DOI:** 10.1002/open.70336

**Published:** 2026-11-06

**Authors:** Hatice Gamze Sogukomerogullari, Celal Tuğrul Zeyrek, İlhan Özer İlhan, Yasemin Sunucu‐Karafakioglu, Sadeq K. Alhag, Musa A. Said, Senem Akkoc

**Affiliations:** ^1^ Department of Medical Services and Techniques Gaziantep University, Vocational School of Health Services Gaziantep Türkiye; ^2^ Uluğ Bey High Technology Application and Research Center (ULUTEM) Gaziantep University Gaziantep Türkiye; ^3^ Ankara University, Institute of Nuclear Sciences Ankara Türkiye; ^4^ Faculty of Sciences Department of Chemistry Erciyes University Kayseri Türkiye; ^5^ Faculty of Education Department of Mathematics and Science Education Uşak University Uşak Türkiye; ^6^ Health Specialties, Basic Sciences and Applications Unit King Khalid University, Applied College Mohayil Asir Abha Saudi Arabia; ^7^ Faculty of Science Department of Chemistry Islamic University of Madinah Madinah Saudi Arabia; ^8^ Faculty of Pharmacy Department of Basic Pharmaceutical Sciences Süleyman Demirel University Isparta Türkiye; ^9^ Faculty of Engineering and Natural Sciences Bahçeşehir University Istanbul Türkiye; ^10^ D’Decus Cosmetics Industry and Trade Limited Company (Sue & Larosel Brands) Isparta Türkiye

**Keywords:** cytotoxic activity, metal complexes, molecular docking, molecular dynamics simulation, ONNO type Schiff base

## Abstract

A Schiff base ligand (H_2_L) was synthesized through the condensation of 3,5‐dibromo‐2‐hydroxybenzaldehyde with 1,2‐phenylenediamine and subsequently complexed with Cu(II), Ni(II), Pt(II), Fe(II), and Pd(II) ions. Synthesized compounds were examined using FT‐IR, UV–Vis, NMR, mass, elemental analysis, molar conductivity, and magnetic susceptibility. The H_2_L and transition metal complexes were tested for their cytotoxic activity potential against human liver carcinoma (HepG2) and lung adenocarcinoma (A549) cell lines. The cytotoxic activities of compounds were determined based on their IC_50_ values. The Pt(II) complex exhibited high cytotoxic activity against both HepG2 and A549 cell lines; the IC_50_ values were obtained as 13.32 and 12.26 µM, respectively. In contrast, the Ni(II) and Fe(II) complexes displayed relatively weaker cytotoxic effects. The geometry of the PtL complex was optimized through density functional theory (DFT) calculations. Molecular docking studies were conducted to elucidate the noncovalent interactions between the optimized PtL complex and the active sites of protein BCL‐2 (PDB ID: 6QGK), selected as a putative molecular target relevant to HepG2 cells, using the DFT optimized geometry of PtL.

## Graphical abstract

A Schiff base ligand and its metal complexes were synthesized and characterized. Compounds were tested in cancer and healthy cell lines. The results demonstrated that Pt complex had the highest antiproliferative activity. The geometry of complex was optimized through density functional theory calculations. Molecular docking studies were conducted.

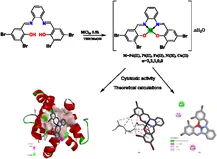

## 1 Introduction

The use of inorganic compounds in medicine dates back over 5000 years, but it is only very lately that these compounds have been developed with a focus on the synthesis of novel anticancer drugs [1]. Cancer develops when cells’ DNA is damaged, leading to their uncontrolled growth and inability to respond normally to the body’s attempts to regulate a particular tissue. Tumors of varying degrees are formed when the afflicted cells proliferate and disperse at a fast pace [2]. Several therapies are available, but platinum‐based drugs, especially cisplatin and carboplatin, have ruled the therapy landscape for many tumors for the last four decades [3, 4, 5]. Recently, metal complexes have garnered the attention of medical chemists [6]. It has been previously proven that complexes initially intended as anticancer medicines have significant efficiency against pathogens [7]. Schiff bases are one of the types of chemical compounds that have been examined extensively and have been shown to have the potential to be used in a variety of fields [8]. Schiff bases serve as exceptional ligands owing to the presence of the azomethine group [8]. The tetradentate Schiff base known as N,N′‐bis(salicylidene)‐1,2‐phenylenediamine (salophene) is composed of two phenolic hydroxyl groups that are coupled with two imine moieties [9]. A significant number of studies have been carried out on *o*‐phenylenediamine‐derived ligands and their complexes for the purpose of bioactivity analysis.

Salen derivatives have emerged as a target for drug research in the context of combating cancer diseases among the diverse array of biologically active metal Schiff base complexes [9]. Since 2005, after the announcement of the synthesis of metal‐based catalytic DNA via the precise integration of metal‐salen complexes into double helices, aimed at creating alternative base pairs for DNA/RNA synthesis with anticipated novel properties, research in this domain has intensified [10]. Recent studies have highlighted the potential of salophen‐type Schiff base metal complexes as promising candidates for anticancer applications. Tetradentate N_2_O_2_ Schiff base complexes have attracted particular interest because metal coordination can significantly influence their structural, electronic, and biological properties [11, 12]. Recent investigations combining cytotoxicity assays with molecular docking have further demonstrated the potential of these complexes for understanding and improving their anticancer activity [12, 13]. These findings emphasize the importance of exploring the relationship between Schiff base structure, metal coordination, and biological activity. However, despite the extensive investigation of salophen‐type metal complexes, systematic comparisons of different metal centers coordinated to the same halogenated tetradentate Schiff‐base framework, together with comparative cytotoxicity and molecular docking studies, remain limited.

DFT calculations for PtL were performed by using the B3LYP/LANLDZ level by using DFT to predict optimized molecular structure. Molecular docking studies can provide complementary computational insight into the potential interactions of novel complexes with biologically relevant targets [14, 15]. Molecular docking constitutes a widely employed computational approach that provides preliminary predictions and visual representations of how such complexes may interact with biological macromolecules, particularly proteins, prior to experimental validation. BCL‐‍2 (PDB ID: 6QGK) has been identified as a pivotal therapeutic target in oncology due to its ability to inhibit apoptosis triggered by diverse stressors, including hypoxia and oxidative damage [16]. In this context, the discovery and development of BCL‐2 inhibitors represent a promising avenue for anticancer drug research. Analysis of the Pt complex against BCL‐2 (PDB ID: 6QGK) enables the assessment of predicted binding affinity and interaction modes, providing a possible molecular rationale for observed cytotoxic activity. However, these computational results can be regarded as hypothesis‐generating rather than evidence of mechanism of action, thereby providing a plausible hypothesis regarding potential protein–ligand or complex interactions that may be relevant to the observed cytotoxic activity.

In this study, the salophen ligand has been synthesized through the reaction of 3,5‐dibromosalicylaldehyde with *o*‐phenylenediamine. This ligand engaged with copper, platinum, palladium, iron, and nickel chloride to produce the respective metal complexes (CuL, PtL, PdL, FeL, and NiL). The ligand and its metal complexes underwent evaluation in cancer cell lines. Through the use of DFT calculations performed, the shape of the PtL complex was optimized throughout the optimization process. To get a better understanding of the noncovalent interactions that occur between the optimized PtL complex and the active sites of protein BCL‐2 (PDB ID: 6QGK), which is a protein that represents the HepG2 cell line, molecular docking experiments were carried out. These studies used the DFT‐optimized geometry of PtL. To contextualize the present findings, a comparative analysis with prior reports was conducted (Table 1). The study was conducted by Behzad et al. [20] investigated transition‐metal complexes against A549 cells, reporting moderate docking scores and cytotoxic effects. Similarly, another study [21] focused on organometallic derivatives targeting kinases, yielding favorable docking results with partial biological validation. In contrast, the present work introduces newly synthesized compounds, further investigated by molecular docking calculations, which exhibited favorable predicted binding energies and interaction profiles toward the selected targets. This highlights the novelty of our approach and underscores the potential of these compounds for therapeutic development.

**TABLE 1:** Comparison of the present study with previously reported related Schiff‐base metal complexes.

References	Schiff‐base ligand/ structural feature	Metal ion, s	Structural characterization	Biological evaluation	A549	Molecular docking	Distinction from the present study
[17]	Related salophen‐type Schiff base ligand	—	Spectroscopic characterization	—	—	—	First report of the Schiff‐base framework
[18]	Same 3,5‐dibromo‐substituted salophen ligand used in the present study	Ni(II)	Spectroscopic/structural characterization	—	—	—	Ni(II) complex was previously reported, but without biological evaluation.
[19]	3,5‐Dichloro‐substituted symmetrical salophen ligand	Ni(II), Cu(II), Zn(II)	Spectroscopic, electrochemical and computational characterization	—	—	✓	Related halogenated salophen system with different ligand substitution and metal ions.
[20]	Structurally different Schiff base ligand	Cu(II)	Spectroscopy and SC‐XRD	Antiproliferative assays	✓	✓	Different ligand architecture and Cu(II) based system; combines A549 cytotoxicity and molecular docking.
[21]	Structurally different Schiff base/mixed‐ligand system	Cu(II)	Spectroscopic/structural characterization	Biological/computational evaluation	✓	✓	Different ligand framework; includes A549 and molecular docking.
Present study	3,5‐Dibromo‐substituted symmetrical salophen ligand	Ni(II), Cu(II), Fe(II), Pt(II), Pd(II)	FT‐IR, UV–Vis, NMR, elemental analysis, molar conductivity, magnetic susceptibility	Antiproliferative activity against HepG2 and A549	✓	✓	Five‐metal comparative study combining biological evaluation and molecular docking.

—, Not reported.

## 2 Experimental Section

### 2.1 General

H_2_L was synthesized for the first time by Zheng et al. [17]. Ligand synthesis was carried out under optimized reaction conditions, differing from the methods reported in the literature. The main difference between this study and the literature is that the reaction was completed at room temperature and in a short time, only 1 h. Thus, a more practical and energy‐efficient synthesis approach has been presented.

### 2.2 Synthesis of Ligand (H_2_L)

The ligand N,N′‐*o*‐phenylene‐bis(3,5‐dibromosalicylideneimine) (H_2_L) was synthesized through a condensation reaction between 1,2‐phenylenediamine (0.1 g, 0.92 mmol) and 3,5‐dibromo‐2‐hydroxybenzaldehyde (0.517 g, 1.84 mmol). The two reactants were dissolved in ethanol, and the resulting mixture was maintained at room temperature under stirring for 1 h (Scheme 1). Upon completion of the reaction, an orange solid was formed in the reaction medium. The precipitated product was collected by filtration and subsequently washed with methanol and diethyl ether. Yield: 92%, m.p.: 211–213 °C. FT‐IR (ATR)*v* cm^−1^: 3066 (C─H)_aromatic_, 2970 (C─H)_aliphatic_, 1610 (C═N). ^1^H NMR (DMSO‐d_6_, 400 MHz): *δ* 14.07 (s, 2H, OH), 8.99 (s, 2H, N═CH), 7.35–7.98 (m, 8H, Ar‐H). UV–vis (DMF, λmax, (Abs.)): 270 (0.501), 290 (0.326), 305 (0.279), 350 (0.209), 455 (0.305) nm; Λ_M_ (10^−3^ M, in DMF, µS/cm): 3.21. LC–MS/MS (*m/z*): 631 [H_2_L]^+^ Anal. Calc. For C_20_H_12_Br_4_N_2_O_2_ (631.94 g/mol), C: 38.01; H: 1.91; N: 4.43%. Found: C: 37.99; H: 1.78; N: 4.36%.

**SCHEME 1:**
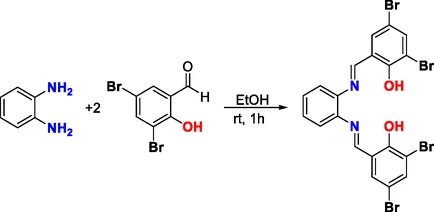
Synthesis of a Schiff base ligand.

### 2.3 Preparation of Transition Complexes of H_2_L

Complex formation was carried out by combining equimolar amounts of the ligand H_2_L (0.125 g, 0.197 mmol) and the corresponding metal salt. Initially, H_2_L was dissolved in a 25 mL of MeOH/THF solvent mixture (1:1, v/v). In parallel, MCl_2_·nH_2_O (0.197 mmol; M═Cu, Ni, Pd, Pt, or Fe; *n* = 2, 0, 0, 0, or 4, respectively) was dissolved in methanol. The metal salt solution was then introduced into the ligand solution, and the resulting mixture was maintained under reflux for 30 min. Upon completion of the reaction, the solid material was isolated by filtration, washed with cold methanol and diethyl ether, and finally dried under reduced pressure to afford the desired metal complex (Scheme 2). The ^1^H NMR, ^13^C NMR, FT‐IR, mass, and UV–vis spectra of compounds are provided in the Supporting Information file (Figures S1–S24).

**SCHEME 2:**
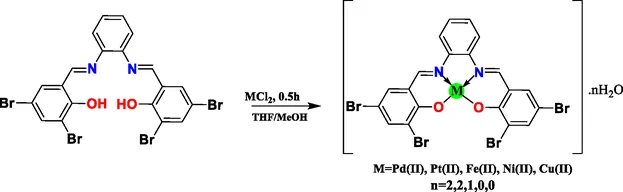
Synthesis of various transition metal complexes.

**[NiL]:** Reddish orange. Yield: 0.106 g (76%). M.p.: 360 °C decomp. FT‐IR (ATR) *v*, cm^−1^: 3057 (C─H)_arom._, 2931 (C─H)_aliph._, 1604 (C═N), 545 (M─N), 482 (M─O). UV–vis (DMF, λmax, (Abs.)): 270 (1.073), 280 (0.738), 300 (0.447), 325 (0.422), 385 (0.638), 455 (0.194), 485 (0.229) nm. ^1^H NMR (400 MHz, DMSO‐*d*_6_) *δ*: 9.01 (s, 2H, ─C**H**═N), 7.89–6.69 (m, 8H, Ar‐**H**). ^13^C NMR (100 MHz, DMSO‐*d*_6_) *δ*: 162.73 (Ar**C** to ─OH), 161.44 (─**C**H═N), 142.59, 139.08, 128.48, 123.62, 122.37, 120.07, 119.25, and 116.82 (ArC). *μ*_eff_: dia. Λ_M_ (10^−3^ M, in DMF, µS/cm): 3.58. LC–MS/MS (*m/z*): 685.6 [M + L − 3H]^+^Anal. Calc. For C_20_H_10_Br_4_N_2_NiO_2_ (688.62 g/mol), C: 34.88; H: 1.46; N: 4.07. Found: C: 34.72; H: 1.35; N: 3.99%.

**[CuL]**: Brown. Yield: 0.100 g (73%). M.p.: 337–338 °C. FT‐IR (ATR) *v*, cm^−1^: 3053 (C─H)_arom._, 2926 (C‐─H)_aliph._, 1604 (C═N), 532 (M─N), 410 (M─O). UV–vis (DMF, λmax, (Abs.)): 270 (0.840), 315 (0.654), 335 (0.554), 350 (0.464), 395 (0.348), 440 (0.535), 480 (0.217) nm; *μ*_eff_: 2.18 BM. Λ_M_ (10^−3^ M, in DMF, µS/cm): 3.22. LC–MS/MS (*m/z*): 694.3 [M + L+ H]^+^anall. Calc. for C_20_H_10_Br_4_CuN_2_O_2_ (693.47 g/mol), C: 34.64; H: 1.45; N: 4.04. Found: C: 34.56; H: 1.33; N: 3.92%.

**[FeL].H**_**2**_ **O (hereafter, FeL)**: Black. Yield: 0.080 g (58%). M.p.: 325 °C decompose. FT‐IR (ATR) *v*, cm^−1^: 3311 (O─H), 3061 (C─H)_arom._, 2991 (C─H)_aliph._, 1595 (C═N), 547 (M─N), 418 (M─O). UV–vis (DMF, λmax, (Abs.)): 270 (0.824), 300 (0.960), 335 (0.633), 370 (0.455), 430 (0.360), 460 (0.255), 490 (0.141) nm; μ_eff_: 5.10. Λ_M_ (10^−3^ M, in DMF, µS/cm): 3.25. LC–MS/MS (*m/z*): 685.9 [M + L − H_2_O]^+^ Aanal Calc. for C_20_H_12_Br_4_FeN_2_O_3_ (703.79 g/mol), C: 34.13; H: 1.72; N: 3.98. Found: C: 33.93; H: 1.68; N: 3.87%.

**[PdL].2H**_**2**_ **O (hereafter PdL)** Cumin yellow. Yield: 0.138 g (91%). M.p.: 322–323 °C. FT‐IR (ATR) *v*, cm^−1^: 3215 (O─H), 3062 (C─H)_arom._, 2975 (C─H)_aliph._, 1612 (C═N), 542 (M─N), 422 (M─O). UV–vis (DMF, λmax, (Abs.)): 270 (0.928), 280 (0.799), 310 (0.498), 325 (0.469), 340 (0.381), 365 (0.255), 420 (0.290), 445 (0.364), 500 (0.155), 580 (0.153) nm. ^1^H NMR (400 MHz, DMSO‐*d*_6_) *δ*: 9.07 (s, 2H, ─C**H**═N), 8.14–7.01 (m, 8H, Ar‐**H**). ^13^C NMR (100 MHz, DMSO‐*d*_6_) *δ*: 159.21 (Ar**C** to ─OH), 153.93 (─**C**H═N), 146.99, 141.73, 137.42, 127.53, 126.84, 122.79, 117.4, and 117.17 (ArC). μ_eff_: dia. Λ_M_ (10^−3^ M, in DMF, µS/cm): 2.01. LC–MS/MS (*m/z*): 773.9 [M + L + H]^+^anall. Calc. for C_20_H_14_Br_4_N_2_O_4_Pd (772.38 g/mol), C: 31.10; H: 1.83; N: 3.63. Found: C: 30.96; H: 1.85; N: 3.70%.

**[PtL].2H**_**2**_ **O (hereafter PtL)** Light brown. Yield: 0.090 g (53%). M.p.: 350 °C decomp. FT‐IR, (ATR) *v*, cm^−1^: 3205 (O─H), 3065 (C─H)_arom._, 2973 (C─H)_aliph._, 1627 (C═N), 545 (M─N), 420 (M─O). UV–vis (DMF, λmax, (Abs.)):270 (1.257), 280 (1.127), 330 (0.406), 385 (0.406), 445 (0.438), 470 (0.657), 575 (0.291), 690 (0.320), 705 (0.394) nm. ^1^H NMR (400 MHz, DMSO‐*d*_6_) *δ*: 9.64 (s, 2H, ─C**H**═N), 8.43–7.29 (m, 8H, Ar‐**H**). ^13^C NMR (100 MHz, DMSO‐*d*_6_) *δ*: 156.36 (Ar**C** to ─OH), 153.76 (─**C**H═N), 143.69, 140.84, 140.09, 128.73, 128.50, 126.96, 119.82, and 118.40 (ArC). μ_eff_: dia. Λ_M_ (10^−3^ M, in DMF, µS/cm): 2.55. LC–MS/MS (*m/z*): 861.4 [M + L]^+^anall. Calc. for C_20_H_14_Br_4_N_2_O_4_Pt (861.04 g/mol), C: 27.90; H: 1.64; N: 3.25. Found: C: 27.76; H: 1.79; N: 3.47%.

### 2.4 Cytotoxic Activity Studies

All equipment and consumables used during the study were sterilized prior to the experiment. DMEM, EMEM, MTT reagent, PBS, trypsin‐EDTA solution, fetal bovine serum (FBS), GlutaMAX supplement, 25 and 75 cm^2^ cell culture flasks, centrifuge tubes, sterile pipettes, cryovials, and 96‐well microplates were obtained from Gibco, Sigma–Aldrich, Thermo Fisher Scientific, and Jet Biofil.

The lung (A549) and liver (HepG2) cancer cell lines were cultured in DMEM containing 10% FBS and 1% GlutaMAX. The mouse fibroblast cell line (L929) was grown in EMEM instead of DMEM. Cells were seeded into 96‐well plates at a density of 5000 cells to well for 24 h. Then, the medium was aspirated and discarded. Fresh medium was then added to wells, and the cells were treated with compounds H_2_L, NiL, CuL, FeL, PdL, and PtL at concentrations of 300, 150, 75, 37.5, and 18.25 µM for 48 h. At the end of this period, the medium in the wells was carefully aspirated, and 50 µL of freshly prepared MTT solution was put to wells. The plates were incubated for 3 h. After incubation, the supernatant was removed, and 200 µL of DMSO was added to wells. The plates were kept in a dark environment under gentle shaking conditions for approximately 30 min to ensure homogeneous dissolution. At the end of this time, the absorbance of the resulting formazan product was measured at 590 nm using an ELISA reader [22, 23]. All experiments were performed independently in triplicate, and the obtained data were expressed as the mean values of the three independent experiments. The results are presented as mean values ± standard deviation (SD). The statistical significance of the experimental data was assessed using GraphPad Prism, version 8.0.2. A one‐way analysis of variance was conducted, followed by Dunnett’s post hoc test for multiple comparisons. Differences were considered statistically significant when the corresponding *p*‐value was below 0.05.

### 2.5 Calculation Details for DFT and Molecular Docking Study

The geometry of PtL was optimized at the B3LYP/LANL2DZ level in the ground state using Gaussian 09, and the resulting structure was subsequently employed in molecular docking studies [24, 25] and visualized by the Gauss‐view program [26]. The calculated bond lengths (Å) and bond and torsion angles (°) of PtL are provided in the Supplementary Data (Table S1). The optimized geometry of PtL is given in Figure 1a. Based on the HOMO (Highest Occupied Molecular Orbital) and LUMO (Lowest Unoccupied Molecular Orbital) values of PtL obtained through DFT/B3LYP/LANL2DZ calculations, the global reactivity parameters were determined. Molecular electrostatic potential (MEP) maps of PtL, which facilitate the interpretation of intra‐ and intermolecular interactions, were also generated at the DFT/B3LYP/LANL2DZ level. Molecular docking studies of the PtL complex were conducted using AutoDock4.2 against the target protein (PDB ID: 6QGK), representative of the HepG2 cell line [27]. The molecular structure corresponding to PDB ID: 6QGK was retrieved from the Protein Data Bank (PDB) and used as the target protein in docking simulations. The protein structure (PDB ID: 6QGK) was processed by removing water and ligand molecules, followed by the addition of polar hydrogens to establish protonation states with Discovery Studio Visualizer 4.0 [28]. The docking input files were generated using AutoDockTools (ADT) [29]. The grid size is defined as 30x30x30 Å with grid center *x* = 10.122, *y* = 1.216, *z* = 2.108, and smooth 0.5 Å for 6QGK. A parameter file named “AD4_parameters.dat” was manually created to define the platinum atom and subsequently input into AutoDock4. The quality of docking results was assessed by calculating the Root mean square deviation between the docked pose and the crystal structure, as well as by evaluating the lowest binding free energy (ΔG) of the most favorable docking pose [30, 31]. Interaction figures were generated using Discovery Studio Visualizer 4.0 [28].

**FIGURE 1:**
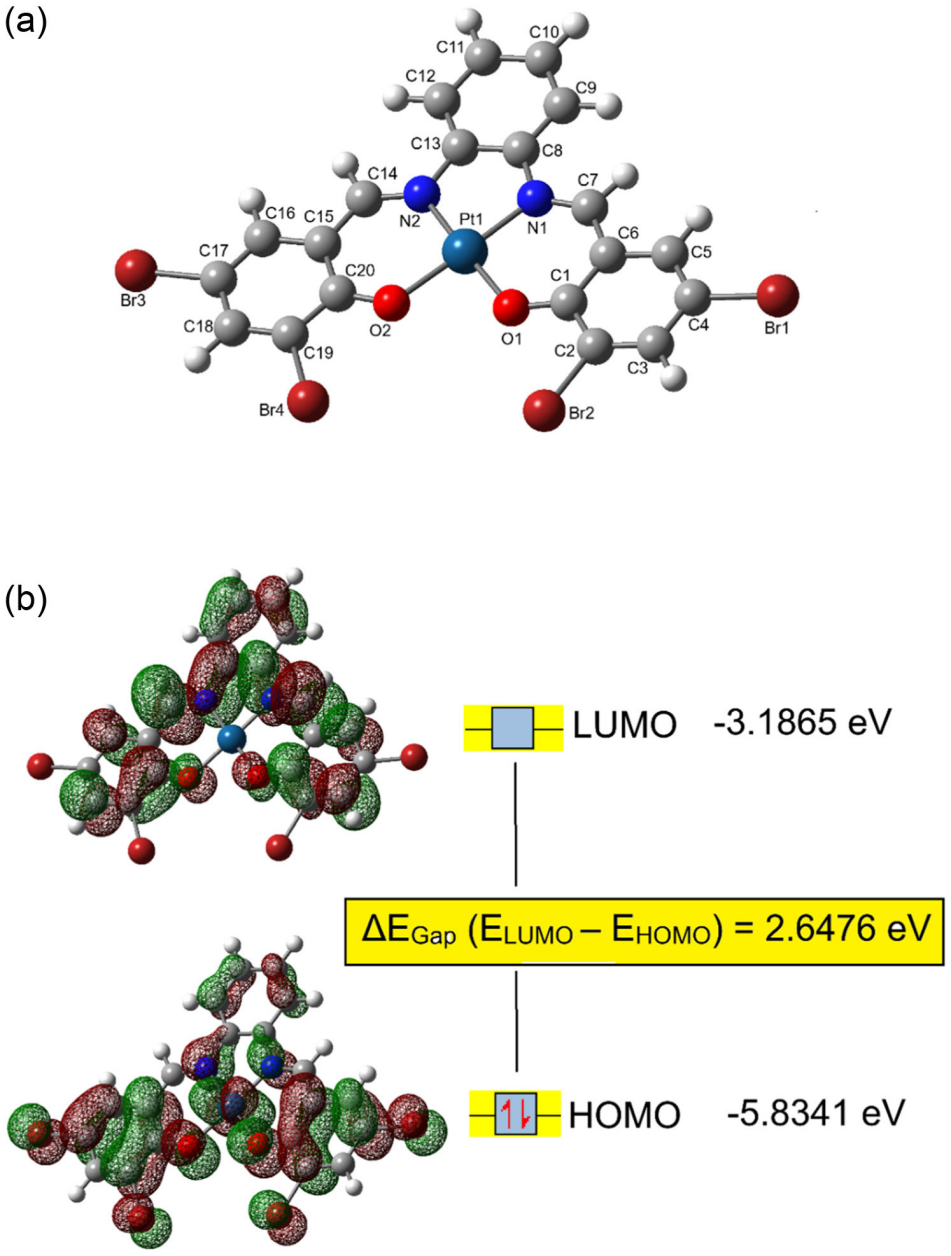
(a) Optimized structure and (b) electron densities surface of HOMOs and LUMOs, energy, and Δ*E*_Gap_ values of PtL.

## 3 Results and Discussion

### 3.1 Characterization of the Ligand and Metal Complexes

The ligand (H_2_L) was first synthesized in 1996 by Zheng et al. by stirring in ethanol at 50–70 °C for 2 h [17]. However, in this study, unlike in the literature, the reaction was completed at room temperature within 1 h. Furthermore, the NiL complex was first reported in the literature by Wei et al. in 2010. The literature states that it was synthesized with a 65% yield by refluxing in ethanol for 2 h. However, in our study, it was synthesized with a 76% yield by refluxing in THF/MeOH for 0.5 h [18]. Metal complexes were formed by combining nickel(II), copper(II), iron(II), palladium(II), and platinum(II) chloride metal salts with the ligand. The synthesized metal complexes were subjected to a comprehensive characterization by elemental analysis, NMR and FT‐IR spectroscopy, UV–vis spectrophotometry, mass, molar conductivity measurements, and magnetic susceptibility analysis. Studies on crystallization were conducted on all substances; however, single crystal formation was unsuccessful. As a result, single‐crystal X‐ray analysis was not feasible. Based on the analytical data, all complexes obtained from the reaction of H_2_L with the corresponding metal salts exhibited a 1:1 metal‐to‐ligand stoichiometry. The electrical conductivities of the complexes were determined in DMF at ambient temperature using 1 × 10^−3^ M solutions. The measurements of molar conductivity within the range of 2.01–3.58 µS/cm suggest that the complexes do not exhibit electrolytic conductivity [32, 33, 34].

^1^H NMR spectra of ligand were acquired. Distinct singlets were observed at approximately *δ* 14.07 and *δ* 8.99 ppm, which are attributed to the phenolic and azomethine protons of the Schiff base, respectively [35]. A multiplet was assigned to the aromatic protons within the range of *δ* 7.98–7.35 ppm in the spectra of ligand. Analyzing the ^1^H NMR spectra of the metal complexes indicates that the ligand engages in coordination through phenolic protons, as demonstrated by the lack of the phenolic proton signal, which is observed as a singlet at *δ* 14.07 ppm in the complex. Upon complexation, the phenolic O─H resonance observed at *δ* 14.07 ppm in H_2_L disappears in the ^1^H NMR spectra of Ni(II), Pd(II), and Pt(II) complexes, consistent with deprotonation of the phenolic groups and coordination of the resulting phenolate oxygen atoms to the metal centers. The azomethine proton is observed at *δ* 9.01, 9.07, and 9.64 ppm for NiL, PdL, and PtL, respectively, compared with *δ* 8.99 ppm for the free ligand. The small shift observed for NiL and the progressively larger downfield shifts observed for PdL and particularly PtL indicate changes in the electronic environment of the azomethine group upon metal coordination and support involvement of the imine nitrogen atoms in coordination.

The aromatic proton resonances are observed over the *δ* 7.89–6.69 ppm range for NiL, *δ* 8.14–7.01 ppm for PdL, and *δ* 8.44–7.29 ppm for PtL. The differences in the chemical shift ranges among the complexes are attributed to changes in the electronic environments of the aromatic rings induced by metal coordination. Overall, the disappearance of the phenolic O─H resonance together with the changes in the azomethine chemical shift provides spectroscopic evidence supporting coordination of the Schiff base ligand through the phenolate oxygen and azomethine nitrogen donor atoms in the Ni(II), Pd(II), and Pt(II) complexes.

The H_2_L spectrum displays bands at 3066 [υ(C─H)_Arom_], 2970 [υ(C─H)_Alp_], and 1610 [υ(C═N)] cm^−1^. In all complexes, the C═N band of the azomethine group, initially observed at 1610 cm^−1^ in the H_2_L ligand, was found within the range of 1627–1595 cm^−1^. This show that the C═N group plays a role in the complexation of H_2_L complexes [36]. The M─N stretching band is indicated by the newly detected bands in the 547–532 cm^−1^ range, whilst the M─O stretching band is denoted by the new bands in the 482–405 cm^−1^ region [37].

UV–visible absorption measurements were performed for the synthesized compounds using DMF as the solvent. The spectra were recorded at ambient temperature over the wavelength interval of 190–800 nm, employing solutions with a concentration of 2 × 10^−5^ M. In the ligands, n–π* transitions are observed in the range of 350–455 nm, while π–π* transitions are noted between 305 and 270 nm [38]. In compounds, n–π* transitions are observed within the range of 325–460 nm, whereas π–π* transitions occur between 315 and 270 nm [39]. Additionally, new absorption bands have been observed in the visible region spectra (470–500 nm) of the complexes compared to the free ligand. These bands are associated with charge‐transfer processes between the ligand and the metal center, namely, ligand‐to‐metal charge transfer or metal‐to‐ligand charge transfer transitions. Specifically, the bands identified in the 575–705 nm range in complexes containing Pt(II) and Pd(II) are associated with d–d transitions. These transitions support the possibility that the complexes in question may have a square‐planar or partially distorted square‐planar geometry (e.g., ^1^A_1g_ → ^1^A_2g_, ^1^A_1g_ → ^1^B_1g_ ) [40, 41, 42, 43].

At 300 K, the FeL complex exhibits an effective magnetic moment of 5.10 *μ*_eff_. This value is consistent with a high‐spin Fe(II) configuration corresponding to *S* = 2. Although the spin‐only magnetic moment expected for an *S* = 2 system is 4.90 BM, orbital contributions arising from spin–orbit coupling can increase the observed value, with magnetic moments in the range of approximately 5.10–5.70 BM being typical [44]. Considering these magnetic data, a square‐planar coordination geometry can be assigned to FeL [45]. For the CuL complex, the measured magnetic moment is 2.18 BM. The magnetic behavior of CuL is compatible with a square‐planar arrangement around the copper center [46]. In contrast, the nickel, palladium, and platinum complexes exhibit diamagnetic behavior, indicating the absence of unpaired electrons in their electronic configurations. Based on these magnetic properties and the supporting spectroscopic and analytical findings, a square‐planar coordination geometry is proposed for all three complexes. This structural arrangement is consistent with the coordination characteristics commonly observed for d^8^‐metal ions in suitable ligand environments [46].

### 3.2 Cytotoxic Activity Studies

Table 2 presents the cytotoxic effects of H_2_L and its various metal complexes on cancer cell lines (HepG2 and A549) and a healthy fibroblast cell line (L929) in terms of IC_50_ (µM) values.

**TABLE 2:** IC_50_ results in two types of cancer and a healthy cell lines.

Compounds	**IC**_**50**_ **,** **µM**		
	HepG2	A549	L929
H_2_L	46.02 ± 3.67	33.53 ± 0.70	20.28 ± 2.57
NiL	125.70 ± 4.50	250.30 ± 6.39	1054.0 ± 5.40
PtL	13.32 ± 1.24	12.26 ± 1.52	24.54 ± 1.35
PdL	73.12 ± 5.26	143.60 ± 6.91	108.10 ± 3.23
FeL	312.40 ± 7.49	130.20 ± 4.73	330.90 ± 6.95
CuL	77.16 ± 3.58	235.70 ± 7.29	980.50 ± 6.86
Cisplatin	35.51 ± 3.87	N.T.*	12.81 ± 2.43

Abbreviation: N.T.*, Not tested.

When the free ligand H_2_L was examined, it was found to have an IC_50_ value of 46.02 ± 3.67 µM in the HepG2 cell line and 33.53 ± 0.70 µM in the A549 cell line. These results reveal that the ligand exhibits a higher cytotoxic effect, particularly in A549 lung cancer cells. When the NiL complex was evaluated, IC_50_ values of 125.70 ± 4.50 µM in HepG2 cells and 250.30 ± 6.39 µM in A549 cells were obtained. These results show that the nickel complex exhibits low cytotoxic activity in both cell lines and is even less effective than the free ligand. It is noteworthy that the activity was significantly reduced, particularly in the A549 cell line.

The platinum complex PtL exhibited the strongest cytotoxic effect in both cell lines. IC_50_ values of 13.32 ± 1.24 µM for HepG2 cells and 12.26 ± 1.52 µM for A549 cells were determined. These low IC_50_ values indicate that the platinum complex has a very high cytotoxic activity and is more effective than all other compounds. This result is consistent with the anticancer activities of platinum‐based compounds. The PdL complex showed IC_50_ values of 73.12 ± 5.26 µM in the HepG2 cell line and 143.60 ± 6.91 µM in the A549 cell line. These data reveal that the palladium complex has a moderate cytotoxic effect but is weaker than the platinum complex. Furthermore, lower activity was observed in A549 cells. The FeL complex exhibited IC_50_ values of 312.40 ± 7.49 µM in the HepG2 cell line and 130.20 ± 4.73 µM in the A549 cell line. The particularly high IC_50_ value in HepG2 cells indicates that this complex has low cytotoxic activity. When the CuL complex was examined, IC_50_ values of 77.16 ± 3.58 µM in the HepG2 cell line and 235.70 ± 7.29 µM in the A549 cell line were determined. These results reveal that the copper complex exhibited moderate cytotoxic effects in HepG2 cells and lower levels of cytotoxic effects in A549 cells.

The IC_50_ values obtained against the L929 healthy fibroblast cell line indicate that the compounds generally exhibit low cytotoxic effects against normal cells. Among the compounds examined, H_2_L (20.28 ± 2.57 µM) and PtL (24.54 ± 1.35 µM) were found to exhibit the highest cytotoxic effects against L929 cells compared to the other compounds. In contrast, the PdL compound (108.10 ± 3.23 µM) has a relatively high IC_50_ value, indicates that this compound exhibits low cytotoxicity toward healthy fibroblast cells. Furthermore, it was observed that the FeL (330.90 ± 6.95 µM), CuL (980.50 ± 6.86 µM), and NiL (1054.0 ± 5.40 µM) compounds were inactive against healthy fibroblast cells. Based on the IC_50_ values given in Table 2, the selectivity index (SI) was calculated by dividing the IC_50_ value in healthy fibroblast cells by the IC_50_ value in cancer cells (Table 3).

**TABLE 3:** Selectivity indexes of compounds.

Compounds	**SI = IC**_**50**_ **(L929) / IC**_**50**_ **(cancer cells)**		
	HepG2	A549	L929
H_2_L	0.44	0.60	1
NiL	8.39	4.21	1
PtL	1.84	2.00	1
PdL	1.48	0.75	1
FeL	1.06	2.54	1
CuL	12.71	4.16	1
Cisplatin	0.36	—	1

When the selectivity indexes between healthy L929 fibroblast cells and HepG2 and A549 cancer cells were evaluated, it was observed that the selectivity of compounds toward cancer cells showed significant differences depending on the compound and the cell line. The H_2_L compound exhibited selectivity indexes of 0.44 and 0.60 against HepG2 and A549 cells, respectively. These values are below 1, indicates that H_2_L has a lower IC_50_ value in L929 healthy fibroblast cells compared to cancer cells and, consequently, does not demonstrate marked selectivity against cancer cells. In contrast, complex NiL exhibited SI values of 8.39 and 4.21 against HepG2 and A549 cells, respectively, demonstrating high selectivity, particularly against HepG2 cells. Complex CuL, meanwhile, exhibited the most striking selectivity among all compounds, with values of 12.71 (HepG2) and 4.16 (A549). This indicates that complex CuL exerts a cytotoxic effect at much lower concentrations in cancer cells compared to healthy L929 cells. Whilst PtL exhibited moderate selectivity with SI values of 1.84 and 2.00 for HepG2 and A549, respectively, the values of 1.48 (HepG2) and 0.75 (A549) indicate limited selectivity against HepG2 and a non‐selective profile against A549 cells.

### 3.3 Chemical Parameters, Frontier MOs, and MEP Analysis of PtL

Frontier molecular orbital (FMO) and MEP analyses of PtL were conducted to elucidate its electronic structure and to provide insights into the intra‐ and intermolecular interactions governing its reactivity. The HOMO and LUMO molecular orbitals of a molecule exhibit distinct contributions and play critical roles in defining its chemical activity. HOMO and LUMO energy values were obtained from the single‐point energy calculation by DFT/B3LYP/LANL2DZ level for PtL, and global reactivity parameters were derived to understand the chemical activity of the molecular structure. For PtL, HOMO and LUMO energy values were obtained from single‐point energy calculations at the DFT/B3LYP/LANL2DZ level, and the corresponding global reactivity parameters were derived to elucidate the chemical activity of the molecular structure. The reactivity descriptors of PtL, including *E*_HOMO_, *E*_LUMO_, energy gap (Δ*E*_Gap_ = *E*_HOMO_ – *E*_LUMO_), ionization potential (*IP*), electron affinity (EA), chemical hardness (*η*), absolute electronegativity (*χ*), and absolute softness (*σ*), are given in Table 4. The DFT/B3LYP/LANL2DZ calculations reveal that PtL exhibits a moderate HOMO–LUMO gap (2.6476 eV), indicating balanced stability and reactivity. Its ionization potential and electron affinity values suggest dual donor–acceptor behavior, while the relatively low hardness and high softness highlight its responsiveness to external interactions. The high electrophilicity index further confirms PtL’s strong tendency to stabilize additional charge, underscoring its pronounced electrophilic character with sufficient structural stability.

**TABLE 4:** The calculated reactivity descriptors of PtL by DFT/B3LYP/LANL2DZ level.

Parameters	PtL
*E*_TOTAL_ (Total energy; Hartree)	−1199.8159
*E*_HOMO_, eV	−5.8341
*E*_LUMO_, eV	−3.1865
Δ*E*_Gap_ (*E*_LUMO_ – *E*_HOMO_: Energy Gap; eV)	2.6476
*IP* (Ionization potential; eV)	5.8341
*EA* (Electron affinity; eV)	3.1865
*η* (Absolute hardness. (IP − EA)/2; eV)	1.3238
*χ* (Electronegativity. (IP + EA)/2; eV)	4.5103
*σ* (Softness. 1/*η*; eV^−1^)	0.7554
*μ*_p_ (chemical potential. −(IP + EA)/2; eV)	−4.5103
*ω* (Electrophilicity. *μ*_p_^2^/2; eV)	13.4652

The frontier orbital distribution of PtL (Figure 1b), obtained at the DFT/B3LYP/LANL2DZ level, reveals that the HOMO is predominantly localized over the aromatic rings and donor atoms, indicating regions of electron density available for nucleophilic interaction. In contrast, the LUMO surface is concentrated around the platinum center and adjacent substituents, demonstrating the preferred sites for electrophilic attack. The moderate energy gap (2.6476 eV) between these orbitals shows a balance between kinetic stability and chemical reactivity. This spatial separation of HOMO and LUMO densities, together with the MEP findings, suggests that PtL exhibits pronounced electrophilic character while maintaining sufficient structural stability to engage in diverse chemical processes. As shown in Figure 1b, the FMOs of PtL display a clear separation in spatial localization and orbital character. The HOMO is largely composed of π‐orbitals delocalized over the aromatic carbon framework and donor atoms (notably nitrogen and oxygen), reflecting electron‐rich regions that can act as nucleophilic centers. In contrast, the LUMO is dominated by antibonding orbitals centered on the platinum atom (Pt1) and extended toward the bromine substituents, designating these sites as favorable loci for electrophilic attack. The calculated HOMO (–5.8341 eV) and LUMO (–3.1865 eV) energies yield a moderate gap of 2.6476 eV, suggesting balanced stability and reactivity. When considered together with the MEP analysis, the observed orbital distribution is consistent with a relatively electrophilic character and provides additional insight into the intrinsic electronic properties and potential reactivity of PtL.

The MEP surface of PtL provides a comprehensive visualization of charge distribution across the complex PtL. As shown in Figure 2, the red regions correspond to areas of negative potential, primarily localized around the oxygen‐ and nitrogen‐donor atoms. These electronegative sites are indicative of high electron density and may serve as preferred regions for hydrogen bonding and electrophilic interactions. In contrast, the blue regions represent positive potential zones, mainly surrounding hydrogen atoms, which are electron‐deficient and thus susceptible to nucleophilic attack. The platinum atom itself exhibits a mixed potential environment, reflecting its role as a coordination center capable of mediating both electron donation and acceptance. The gradient between red and blue areas highlights the polarity within the ligand framework, indicating the presence of multiple potential sites for noncovalent interactions. Overall, the MEP analysis indicates that the electronic distribution of PtL is highly anisotropic, with distinct reactive sites that may influence its chemical and biological activity.

**FIGURE 2:**
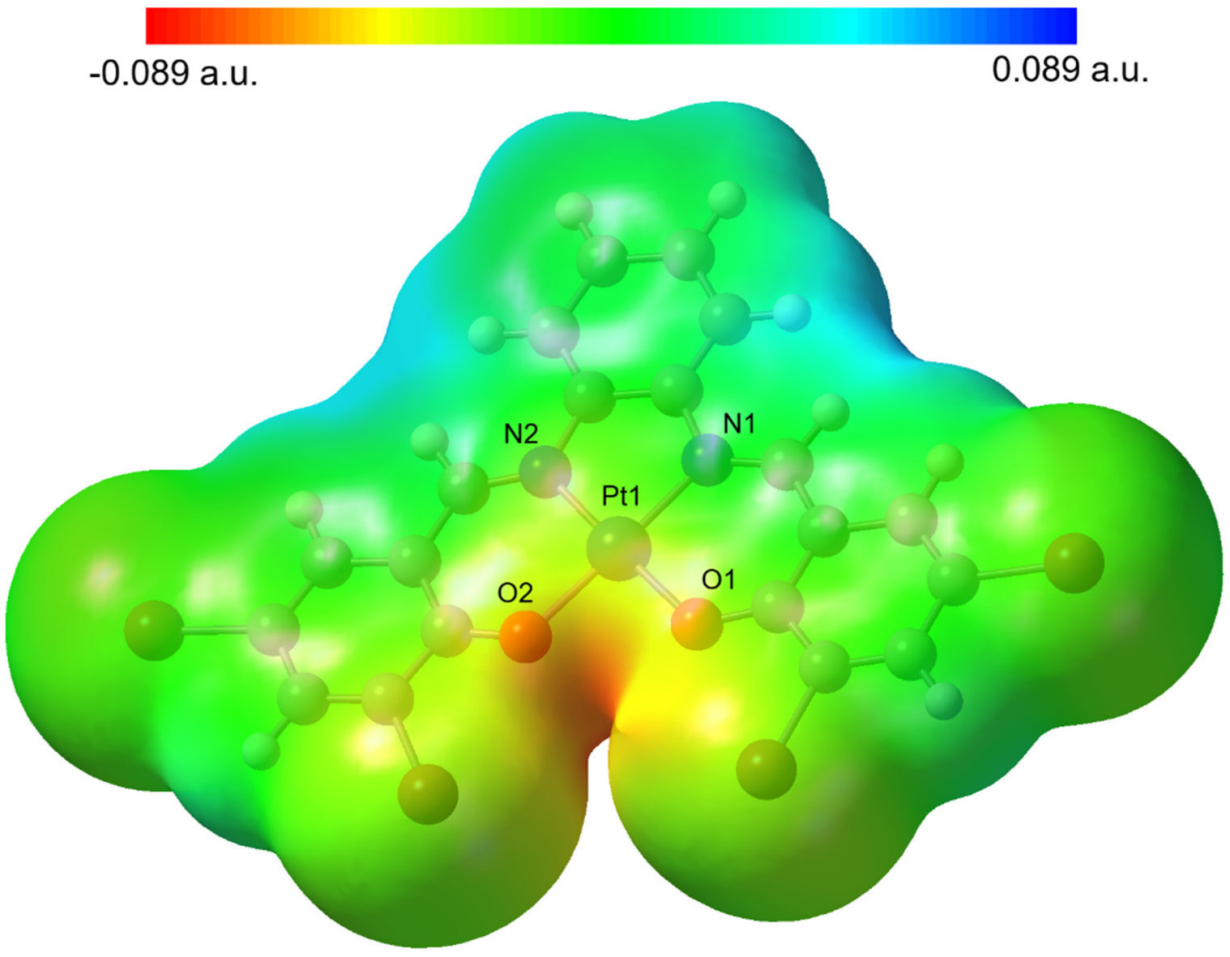
MEP surfaces of compound PtL.

### 3.4 Molecular Docking Studies of PtL Against Target Protein

The biological evaluation results clearly demonstrated that the PtL complex exhibited the strongest cytotoxic activity among all synthesized compounds, with IC_50_ values of 13.32 µM in HepG2 cells and 12.26 µM in A549 cells. This pronounced activity justified the selection of PtL as the primary candidate for molecular docking studies. Thus, focusing on PtL allowed us to correlate the most potent biological response with detailed computational insights. The docking analysis against BCL‐2, a protein overexpressed in HepG2 cells and central to apoptosis regulation, provided computational insights into a possible molecular basis of observed antiproliferative activity. This targeted approach allowed the computational study to be evaluated the experimental findings, showing PtL as the most promising candidate. The DFT‐optimized geometry of PtL provided a reliable structural basis for docking simulations, thereby enhancing the accuracy of predicted binding interactions with the target protein. Figure 1a depicts the optimized geometry of PtL in relation to the target protein (PDB ID: 6QGK), demonstrating the structural framework used for docking analysis. The suggestion that PtL exerts its anticancer activity through inhibition of BCL‐2 is based on computational predictions and the well‐established role of BCL‐2 in apoptosis regulation within HepG2 cells. However, no apoptosis assays, reactive oxygen species measurements, or caspase activation studies were performed in the present work to confirm this mechanism. Therefore, the docking results should be interpreted as supportive evidence rather than definitive proof of the biological pathway. To address this limitation, we have moderated the discussion to emphasize that the computational findings provide a plausible mechanistic hypothesis, which requires further experimental validation through apoptosis‐related assays.

The docking results indicated that PtL interacts favorably with the active sites of protein 6QGK, as predicted by AutoDock4.2. These computational findings suggest a possible interaction with the target protein but do not constitute direct evidence of potential inhibitory activity or mechanism or HepG2 cells [27].

Table 5 lists the interaction parameters, including binding sites, binding types, and bond distances, which illustrate the nature of the docking interactions. Figure 3 depicts the optimal docking orientation of PtL with protein 6QGK, which represents the most stable binding conformation. Calculation of the energetics revealed that the most appropriate docking pose for PtL and protein 6QGK exhibited a relative binding energy and the inhibition constant value of were −6.48 kcal/mol and 17.85 μM, respectively, suggesting a stable binding pose within the docking model.

**TABLE 5:** Binding energy, interactions, and inhibition constant of PtL‐6QGK.

Compound	Least binding energy in kcal/ mol	Inhibition constant (Ki)	Active site residues involved in interactions	Interaction residues distance	Å
PtL	−6.48	17.85 μM	ALA206, ALA207, TYR202	[A:ALA207:N ‐UNK0:Br]^a^ [A:ALA207 ‐ UNK0:Br]^b^ [A:ALA206 ‐ UNK0:Br]^b^ [A:ALA206 ‐ UNK0:Br]^b^ [UNK0 ‐ A:ALA206]^c^ [UNK0 ‐ A:ALA206]^c^ [A:ALA206 ‐ UNK0]^c^ [A:TYR202 ‐ UNK0]^c^ [A:TYR202 ‐ UNK0]^d^	3.3965 3.7032 3.6946 3.9449 5.0121 5.2917 4.5294 5.0086 4.8743

a Conventional hydrogen bonding.

b Alkyl.

c π‐alkyl.

d π–π T, shaped.

**FIGURE 3:**
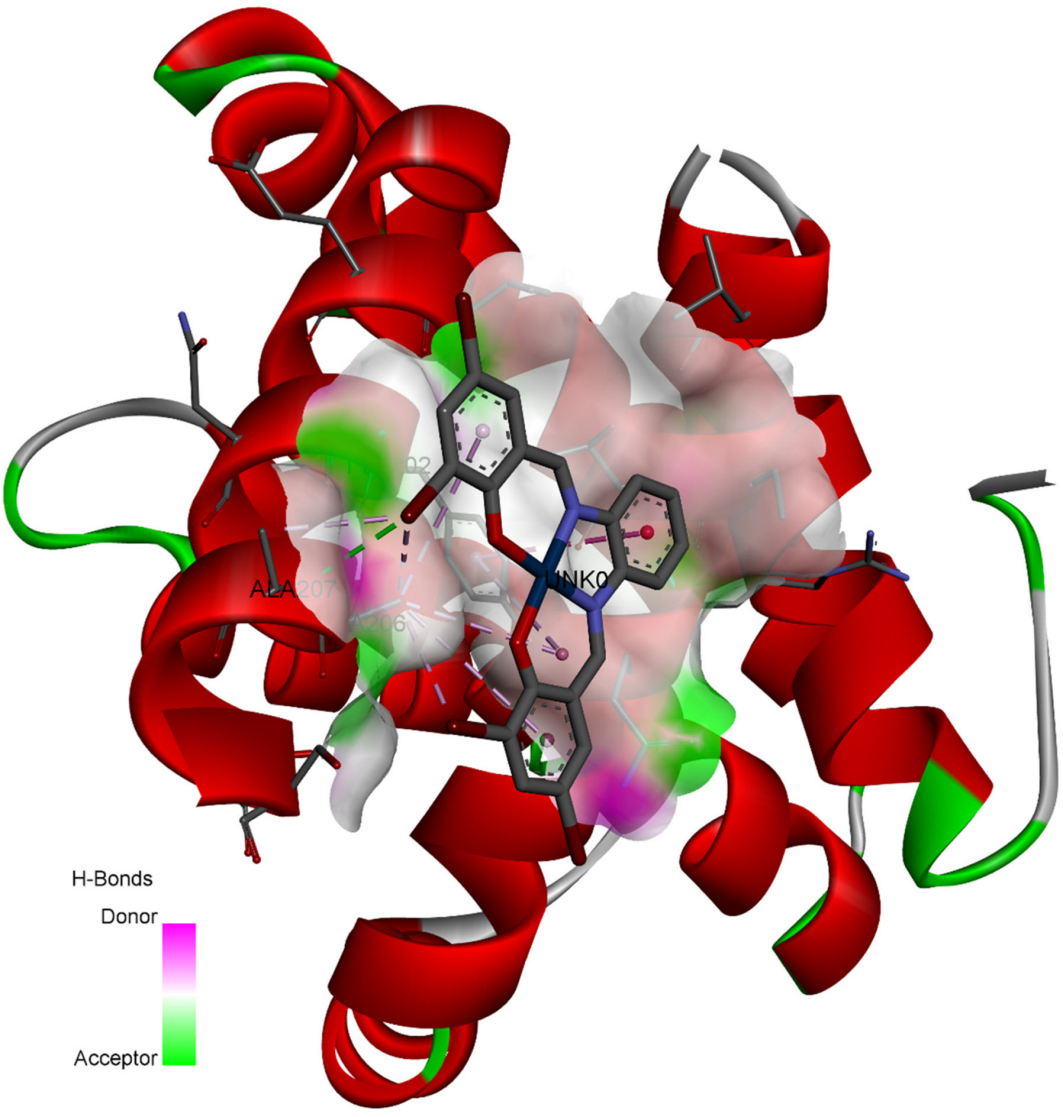
The optimal docking orientation of PtL with protein 6QGK.

Figure 4 illustrates in 3D and 2D the noncovalent binding interactions of PtL with the active site residues of the 6QGK, showing the molecular recognition pattern obtained from the docking model. Molecular docking analysis revealed that PtL engages in interactions with the catalytic sites of amino acids ALA206, ALA207, and TYR202 within the 6QGK. The interaction landscape, presented in Table 5, reveals a network of non‐covalent forces, including one hydrogen bond, four π–alkyl contacts, three alkyl interactions, and one π–π T‐shaped contact, collectively contributing to the overall binding stability. The structural analysis underscores that Br1 of PtL is implicated in conventional hydrogen‐bond formation with the N atom of ALA207, thereby reinforcing the molecular docking pose stability of the complex. The conventional hydrogen‐bond length is 3.3965 Å. Docking simulations identify Br1 atom of PtL as an important interaction site within the predicted protein–ligand complex; however, its contribution to the biological activity of PtL requires further experimental validation.

**FIGURE 4:**
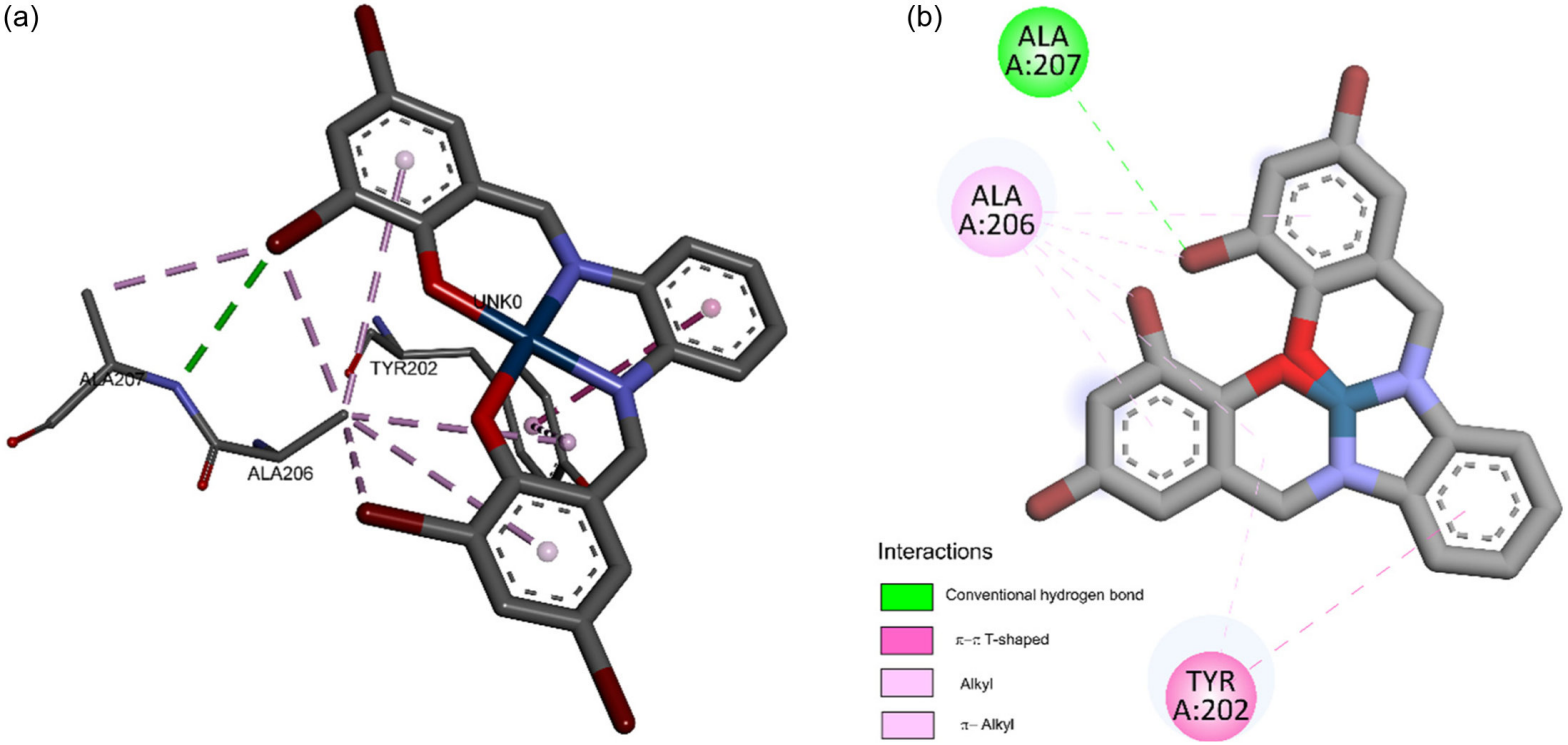
(a) The noncovalent interactions 3D and (b) 2D interaction graph of PtL in the active sites of 6QGK.

While Table 5 provides the numerical docking parameters such as binding energy, inhibition constant, and bond distances, the biological implications of these interactions are particularly noteworthy. The engagement of PtL with residues ALA206, ALA207, and TYR202 within BCL‐2 suggests a potential interaction with the protein and provides a basis for further investigation of its possible biological effects. The hydrogen bond formed between Br1 of PtL and the N atom of ALA207 (3.3965 Å) may contribute to the stability of the predicted binding pose and identifies Br1 as a critical reactive center within docking model. The π–alkyl and π–π contacts further stabilize the predicted ligand–protein association, supporting the possibility of favorable interaction between PtL and target protein. Importantly, these docking interactions align with the strong cytotoxic activity observed experimentally (IC_50_ = 13.32 µM in HepG2 and 12.26 µM in A549), suggesting that the computational findings are consistent with the observed biological activity of PtL. Thus, the docking analysis provides a computational perspective on potential protein–ligand complex interactions and generates hypotheses that may be explored in future mechanistic studies.

The molecular docking studies demonstrated that the most favorable binding pose of PtL with protein 6QGK exhibited a relative binding energy of −6.48 kcal/mol and an inhibition constant (Ki) of 17.85 μM. These values show that the complex establishes a moderately favorable interaction with the predicted binding site residues of 6QGK, representing the HepG2 cell line. The negative binding energy reflects a stable ligand–protein association, while the micromolar Ki value is consistent with a potentially favorable protein–ligand complex interaction within the docking model [47, 48]. Although not in the nanomolar range typical of high‐affinity inhibitors, the observed parameters highlight PtL as a promising candidate with measurable biological activity, warranting further optimization and experimental validation. Accordingly, the docking results should be interpreted as a computationally derived hypothesis regarding potential protein‐ligand or complex interactions rather than as confirmation of the biological mechanism responsible for the observed anticancer activity.

## 4 Conclusion

The present study demonstrates that coordination of the tetrabrominated Schiff base ligand with Ni(II), Cu(II), Fe(II), Pt(II), and Pd(II) ions can substantially influence the biological properties of the resulting complexes. Among the investigated compounds, Pt(II) complex showed the most pronounced cytotoxic activity against both HepG2 and A549 cells, showing the potential role of metal coordination and the platinum center in modulating the biological response of Schiff base complexes. Other complexes, particularly Ni(II) and Fe(II), exhibited relatively weak cytotoxic activity. The docking analysis shows that the Br1 atom of PtL may contribute to the stability of the predicted binding pose through a conventional hydrogen bond interaction with ALA207, while the network of non‐covalent interactions appears to contribute to favorable protein–ligand complex association within the docking model. The molecular docking analysis indicated a favorable predicted interaction between PtL and protein 6QGK, with a relative binding energy of −6.48 kcal/mol and an inhibition constant of 17.85 μM. These computational and experimental findings provide a plausible hypothesis regarding protein‐ligand interactions and support further experimental studies aimed at clarifying the molecular basis of observed biological activity.

## Author Contributions

**Hatice Gamze Sogukomerogullari:** project administration, data curation, formal analysis, investigation, methodology, validation, Conceptualization. **Celal Tuğrul Zeyrek:** software, visualization, methodology, and investigation. **İlhan Özer İlhan:** conceptualization, funding acquisition. **Yasemin Sunucu‐Karafakioglu**: investigation, supervision, formal analysis, methodology, writing–original draft, writing – review and editing. **Sadeq K. Alhag**: funding acquisition, investigation. **Musa A. Said**: investigation, methodology, conceptualization. **Senem Akkoc:** project administration, supervision, data curation, formal analysis, funding acquisition, conceptualization, investigation, methodology, writing–original draft, visualization.

## Funding

This study was supported by Süleyman Demirel Üniversitesi (TBY‐2020‐8053), Erciyes Üniversitesi (FHD‐2022‐11981), and King Khalid University (R.G.P.2/7/47).

## Conflicts of Interest

The authors declare no conflicts of interest.

## Supporting information


^1^H NMR, ^13^C NMR, FT‐IR, and UV‐vis spectra of the synthesized compounds are provided in supporting information file.

## Data Availability

Data will be made available on request.
