# Supplementary material for: Cytotoxic Activity and Molecular Docking Studies of ONNO Type Salophen Schiff Base Metal Complexes
Source: ChemistryOpen. 2026 Nov 6;15(11):e70336. doi: 10.1002/open.70336 (PMC13741202; doi:10.1002/open.70336)
Supplement: Supplementary file 1 [file OPEN-15-e70336-s001.pdf]

Cytotoxic Activity and Molecular Docking Studies of ONNO Type Salophen Schiff Base Metal Complexes

Hatice Gamze Sogukomerogullari[1], Celal Tuğrul Zeyrek[2], İlhan Özer İlhan[3], Yasemin Sunucu-Karafakioglu[4,*], Sadeq K. Alhag[5], Musa A. Said[6], Senem Akkoc[7,8,9,*]

[1]*Department of Medical Services and Techniques, Vocational School of Health Services, Gaziantep University, Gaziantep, Türkiye*

[2]*Institute of Nuclear Sciences, Ankara University, Beşevler, 06100, Ankara, Türkiye*

[3]*Department of Chemistry, Faculty of Sciences, Erciyes University, Kayseri, Türkiye*

[4]*Faculty of Education, Department of Mathematics and Science Education, Uşak University, Uşak, Türkiye*

[5]*King Khalid University, Health Specialties, Basic Sciences and Applications Unit, Applied College, Mohayil Asir Abha, 61421, Saudi Arabia*

[6]*Department of Chemistry, Faculty of Science, Islamic University of Madinah, Madinah 42351, Saudi Arabia.*

[7]*Department of Basic Pharmaceutical Sciences, Faculty of Pharmacy, Süleyman Demirel University, Isparta, Türkiye*

[8]*Faculty of Engineering and Natural Sciences, Bahçeşehir University, Istanbul, Türkiye*

[9]*D'Decus Cosmetics Industry and Trade Limited Company (Sue & Larosel Brands), SDÜ East Campus, Çünür District, 102nd Street, No: 252, Floor: 2, Center, Isparta, Türkiye*

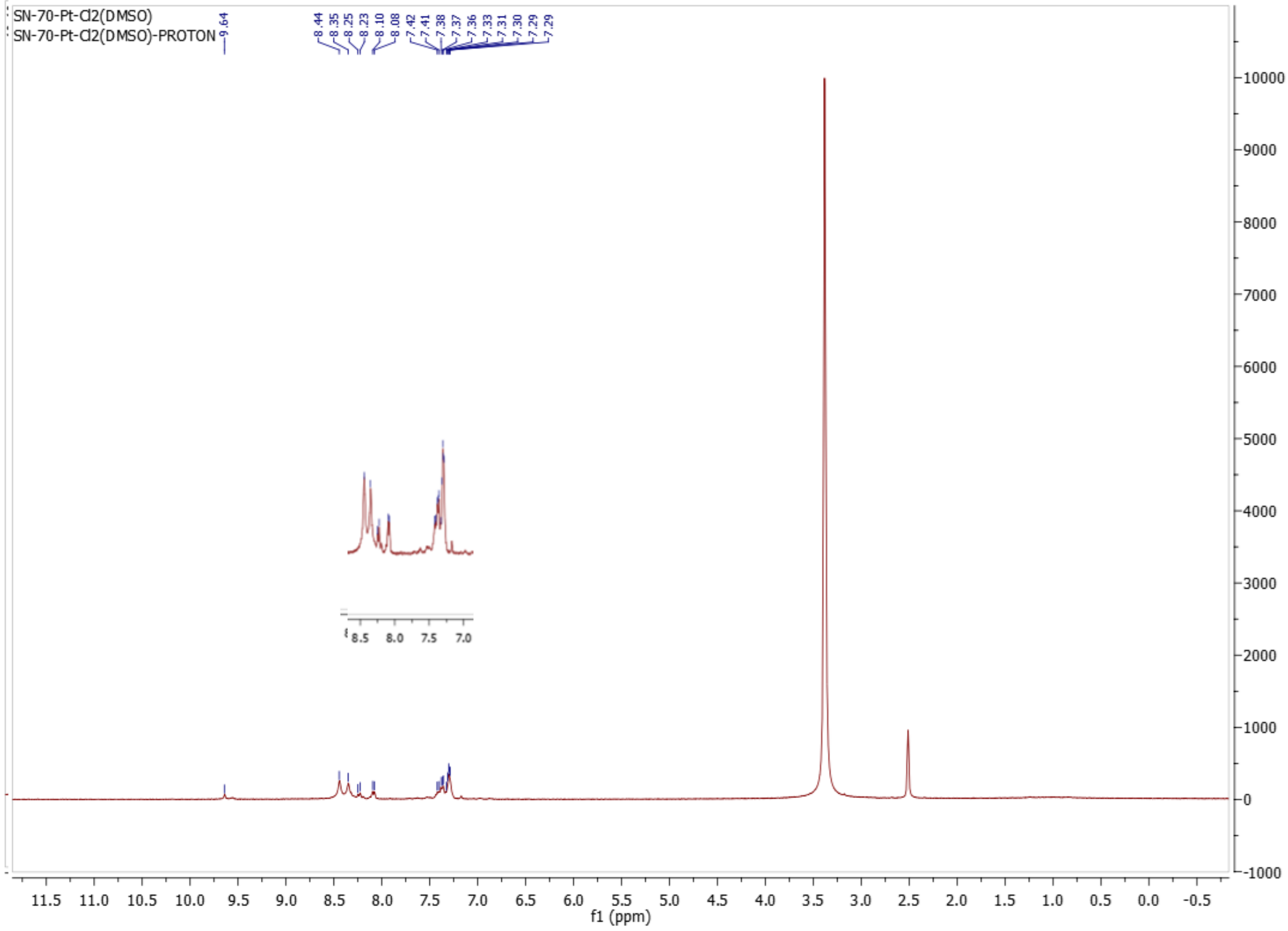

Figure S1. ^{1}H-NMR spectrum of PtL

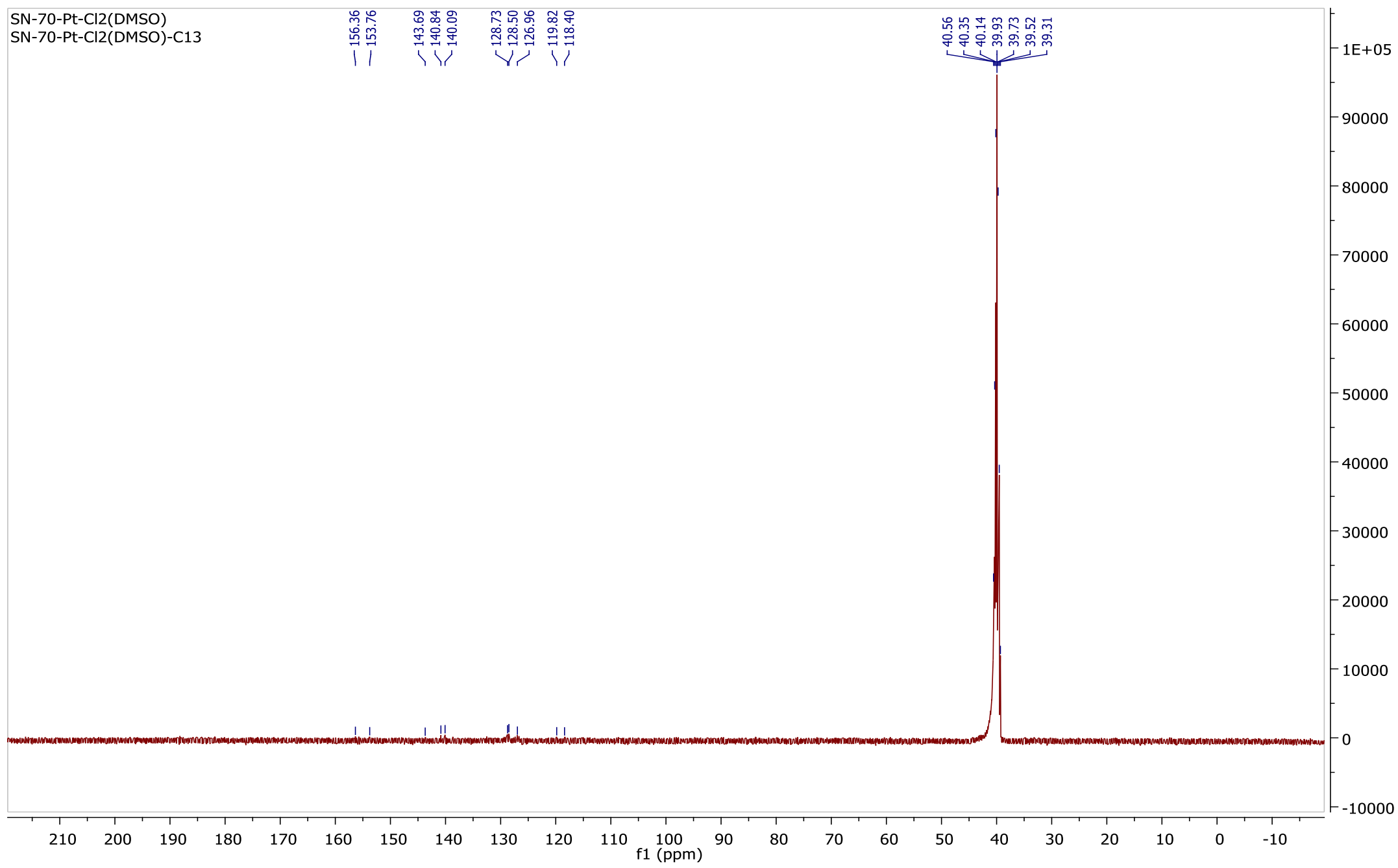

Figure S2. ^{13}C-NMR spectrum of PtL

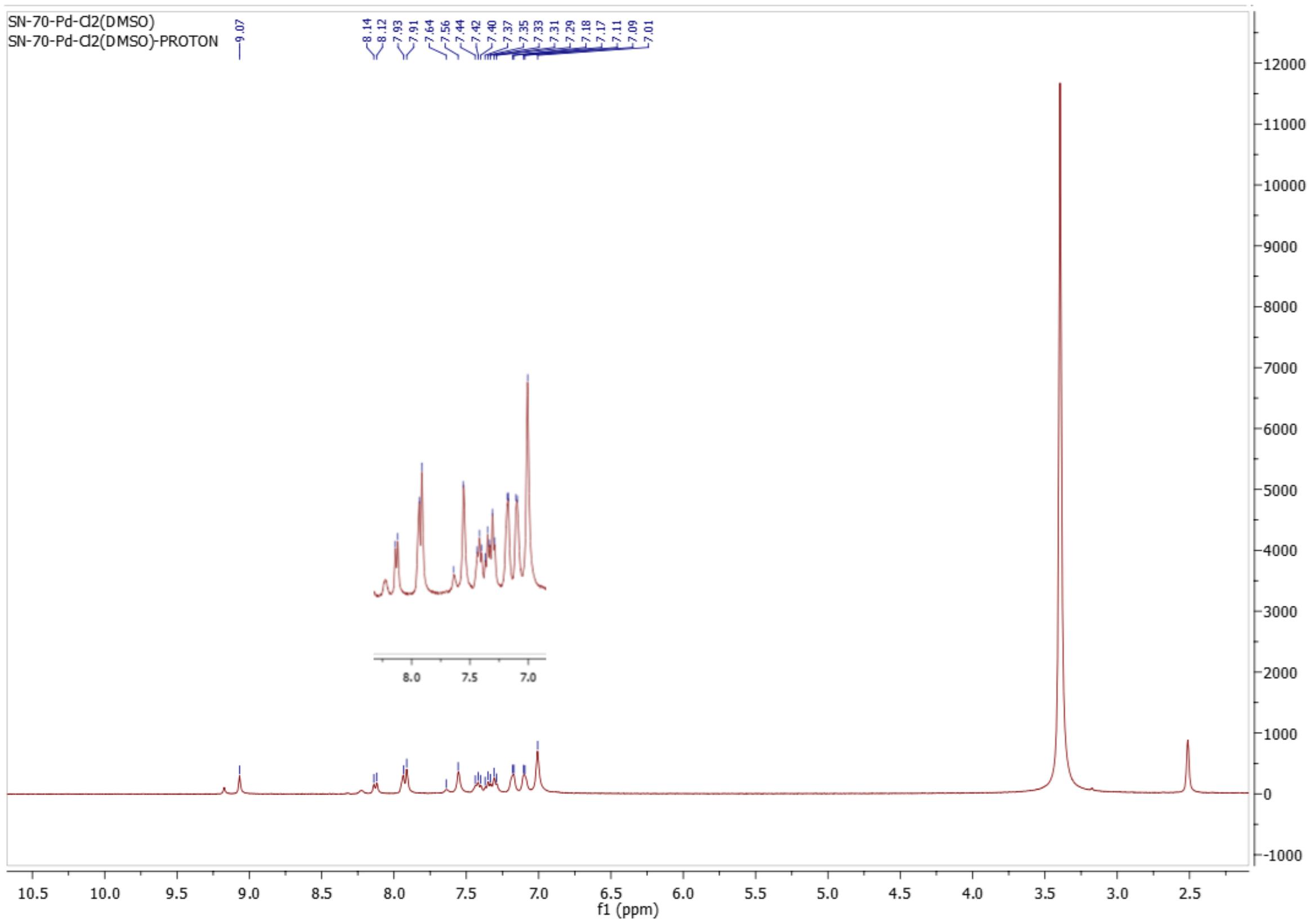

Figure S3. ^{1}H-NMR spectrum of PdL

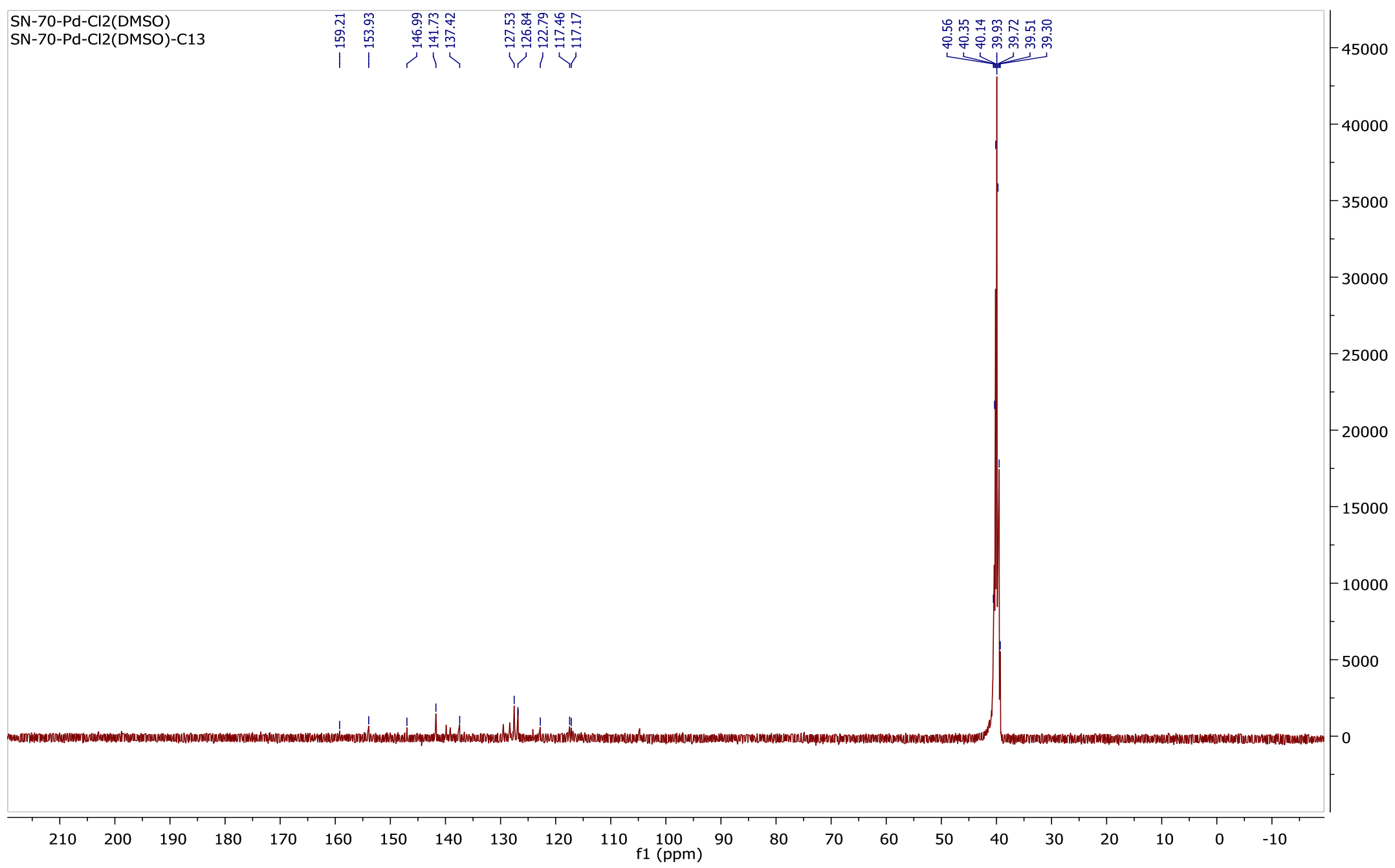

Figure S4. ^{13}C-NMR spectrum of PdL

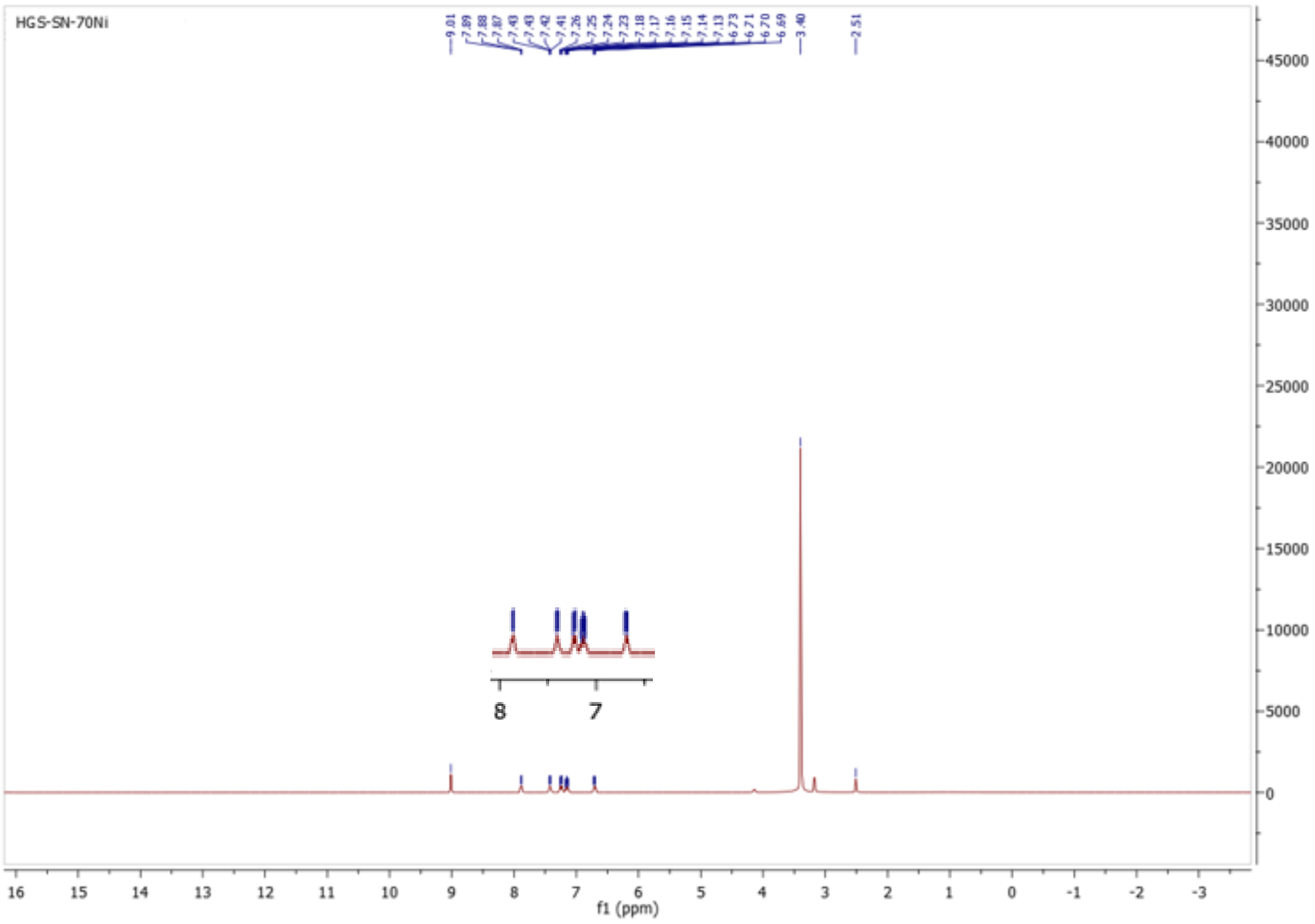

Figure S5. ^{1}H-NMR spectrum of NiL

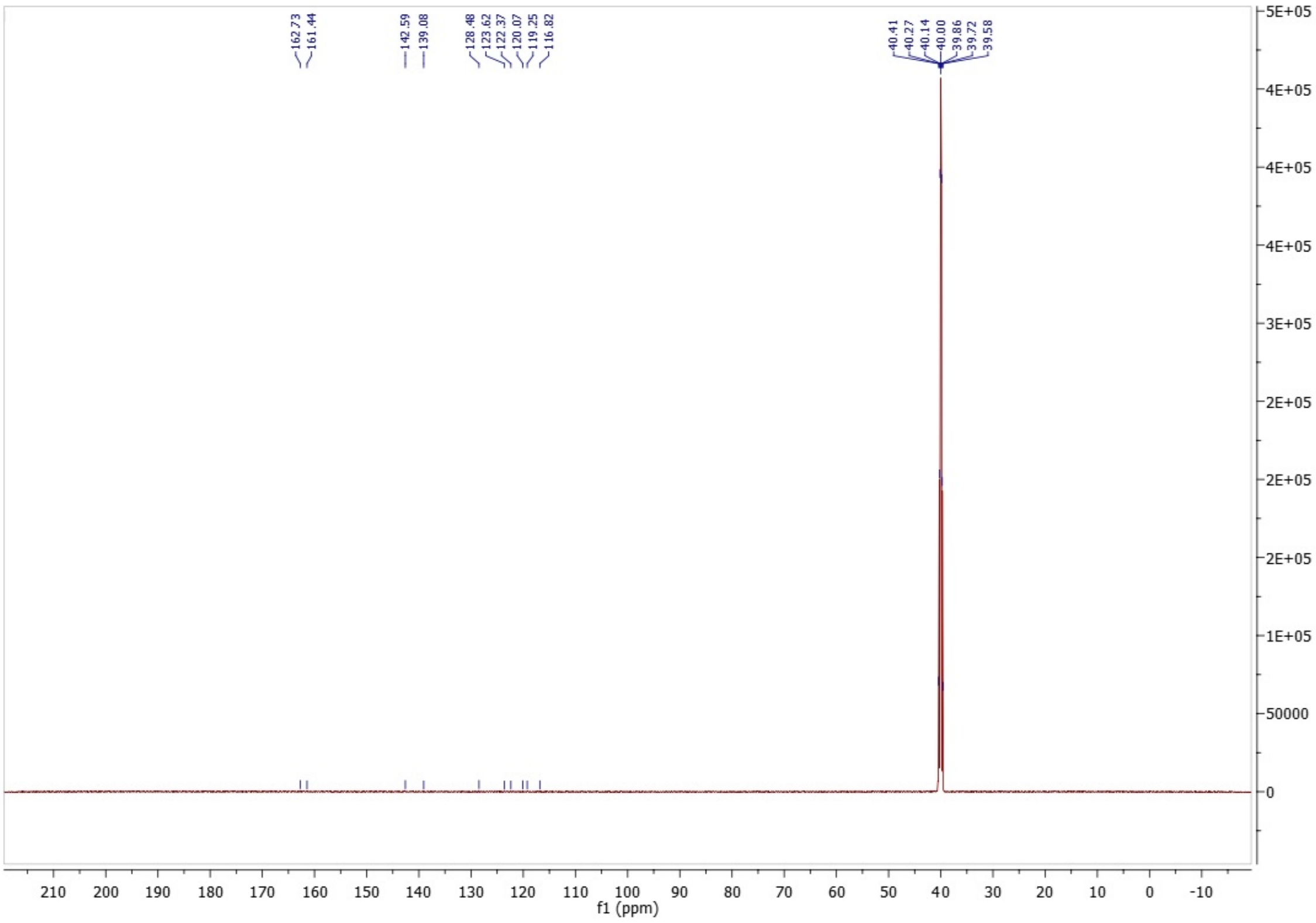

Figure S6. ^{13}C-NMR spectrum of NiL

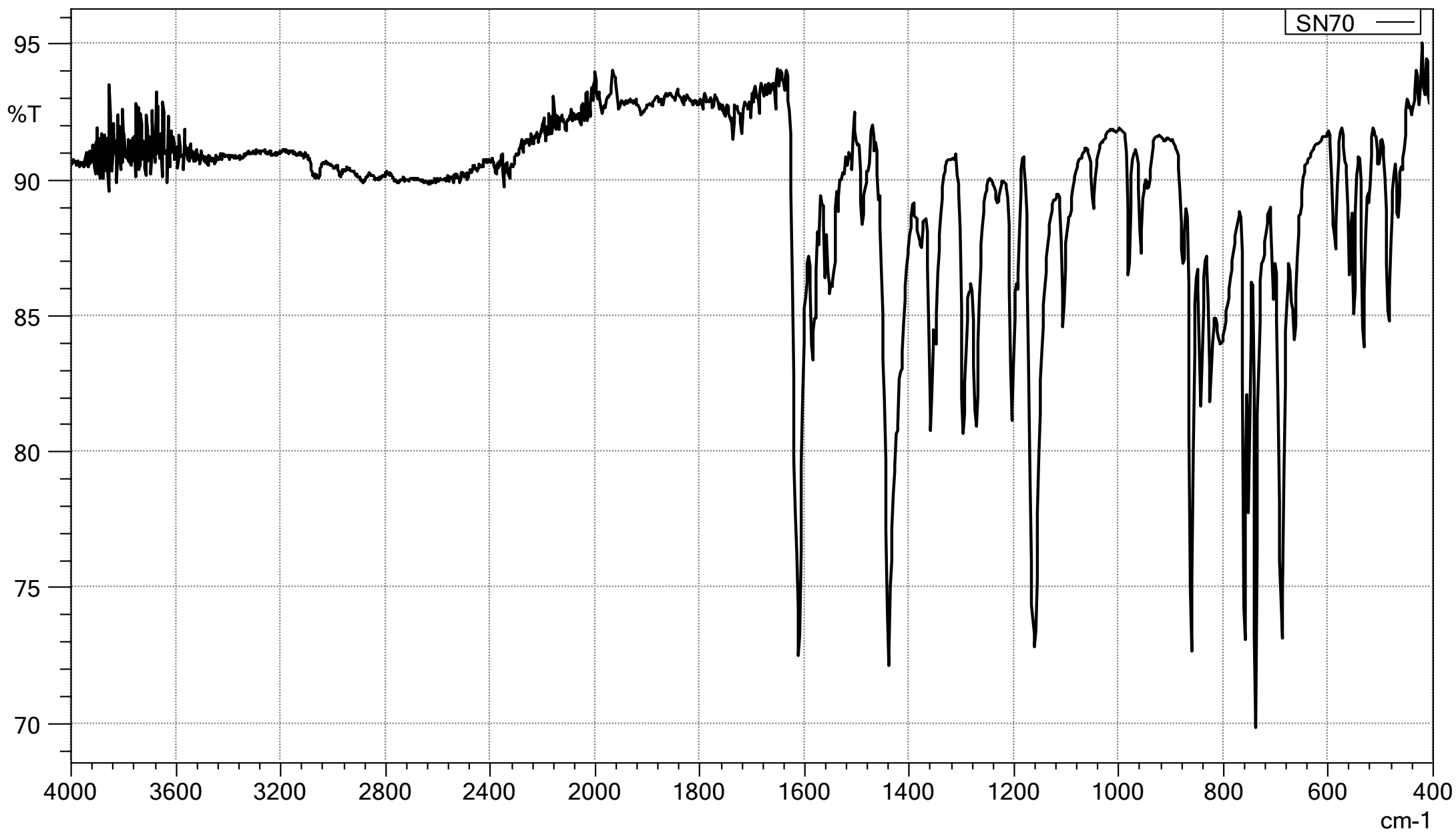

Figure S7. FT-IR spectrum of H_2L

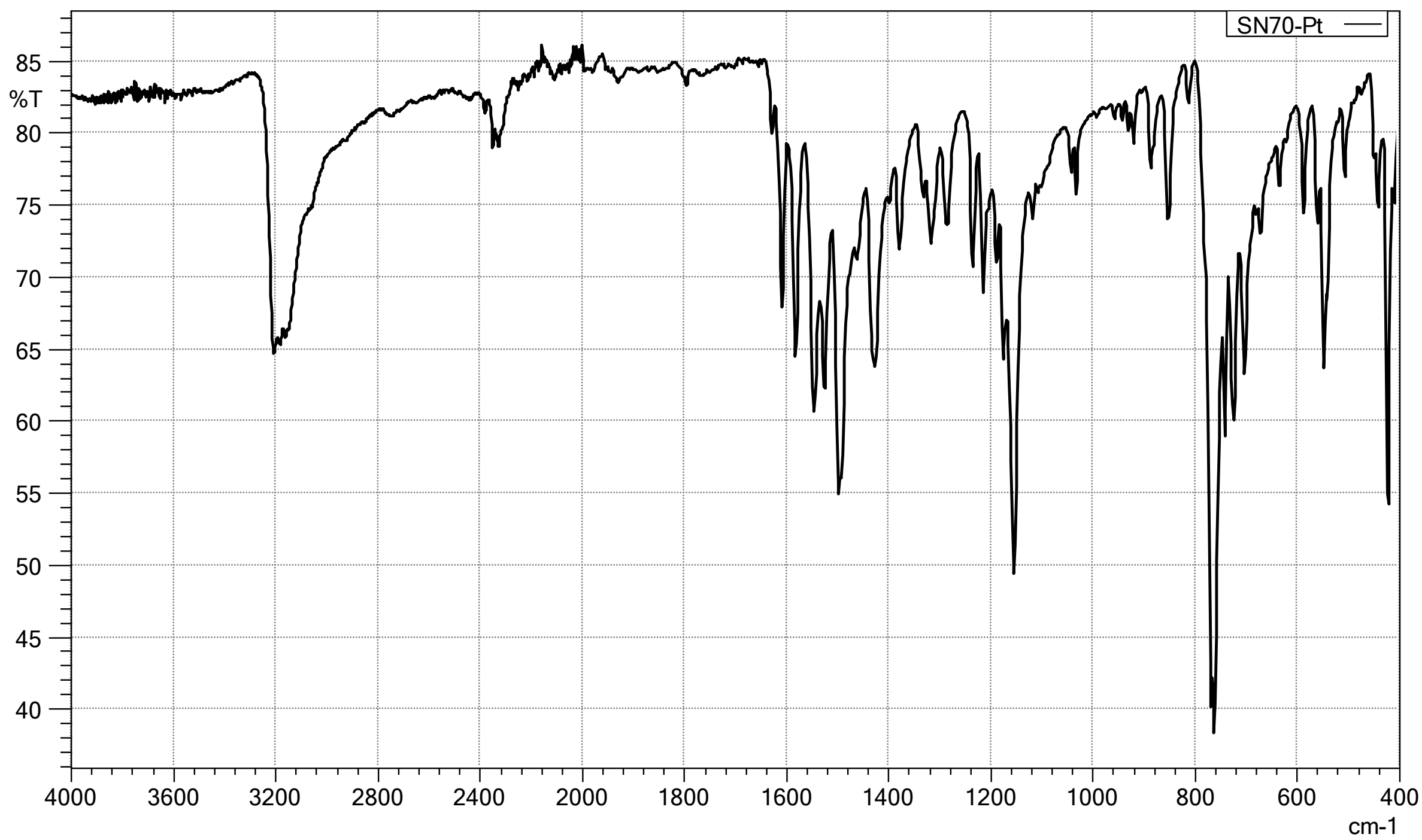

Figure S8. FT-IR spectrum of PtL

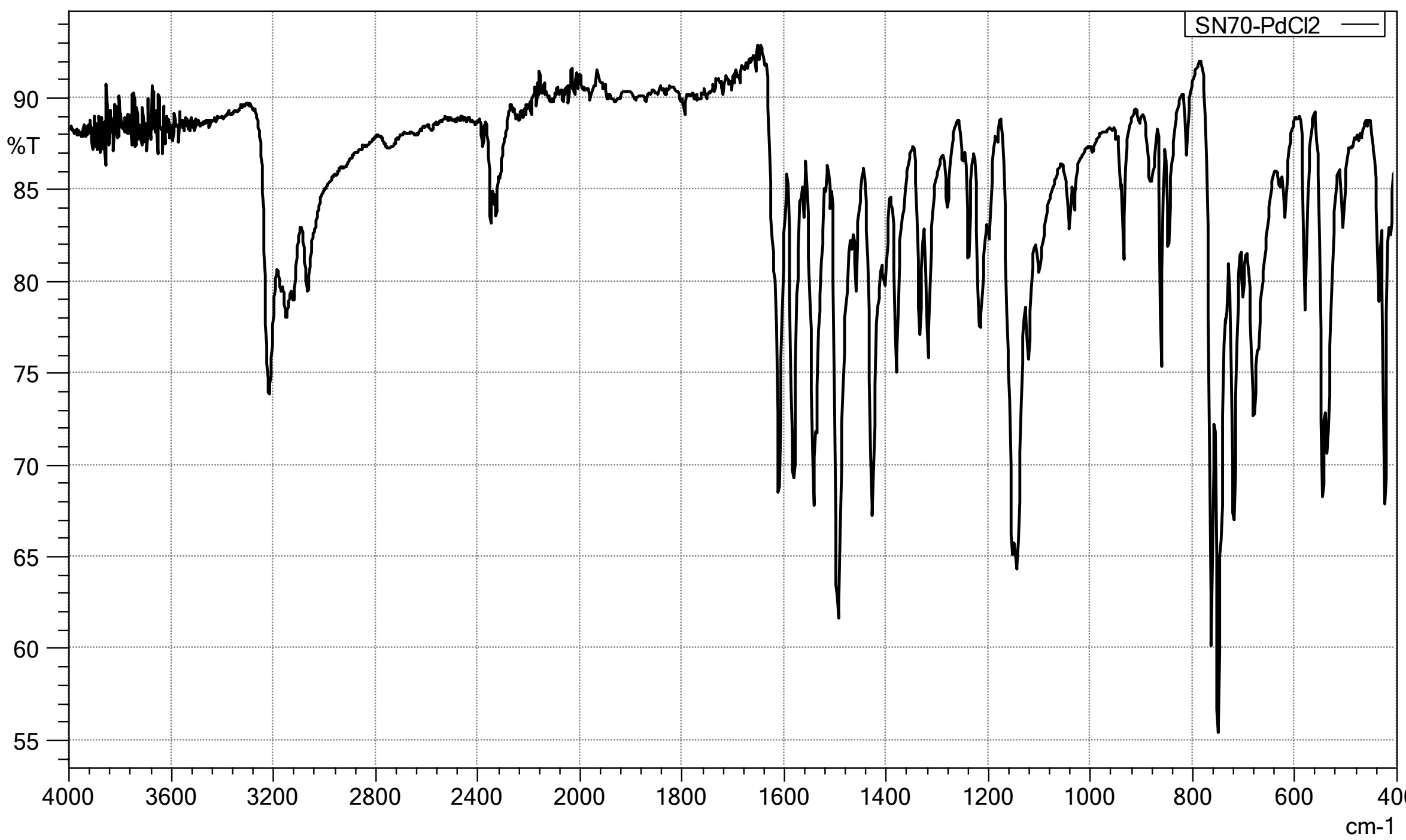

Figure S9. FT-IR spectrum of PdL

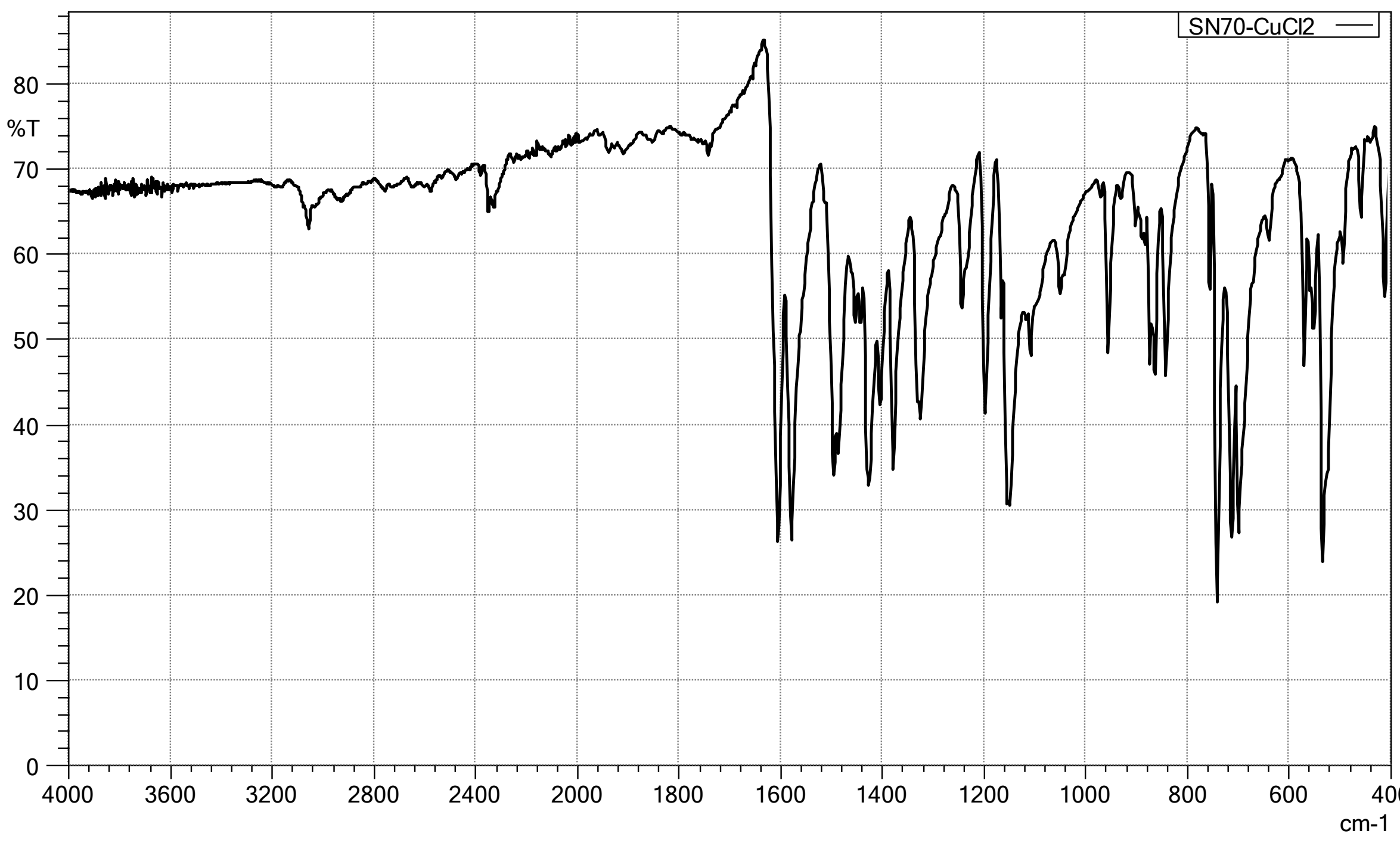

Figure S10. FT-IR spectrum of CuL

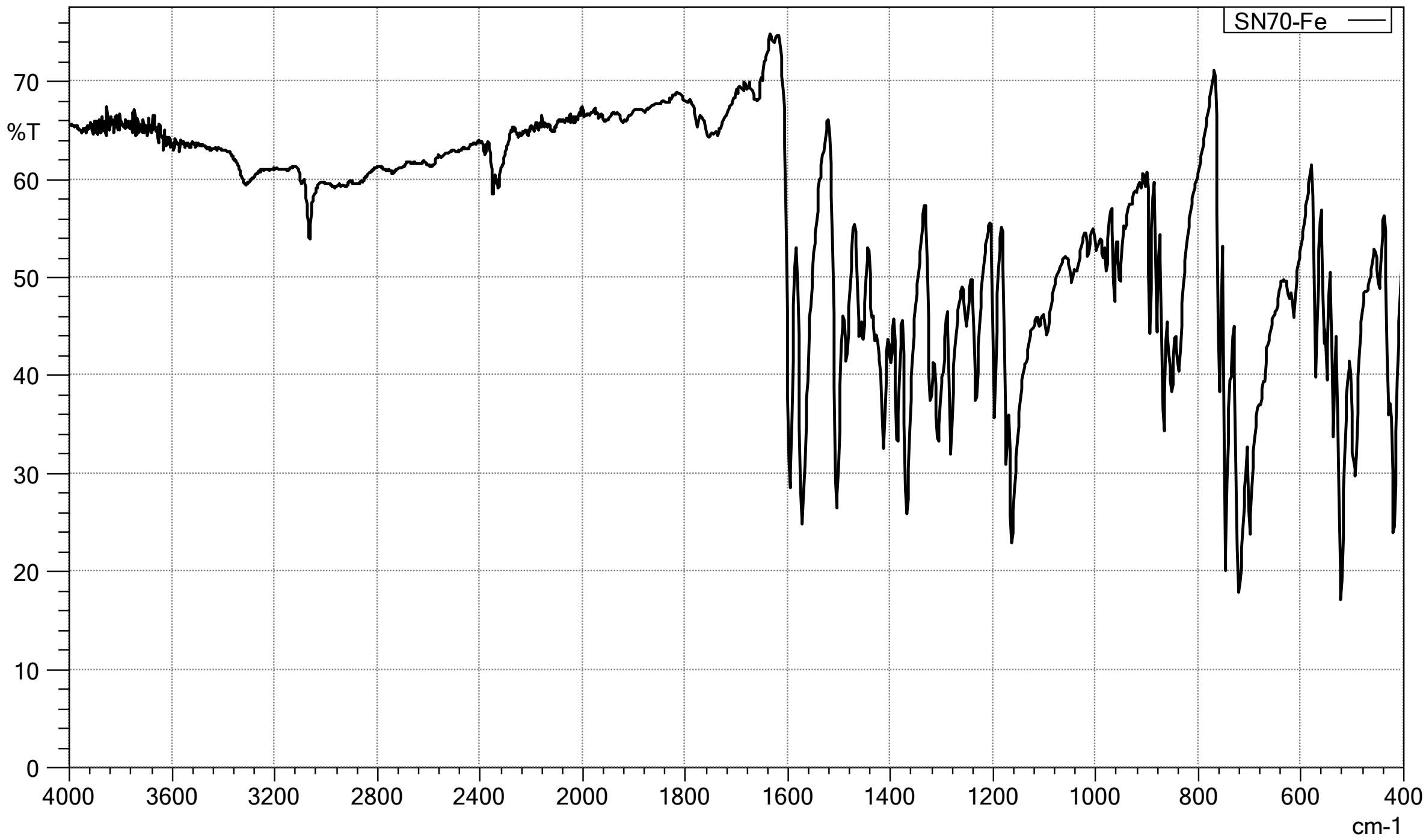

Figure S11. FT-IR spectrum of FeL

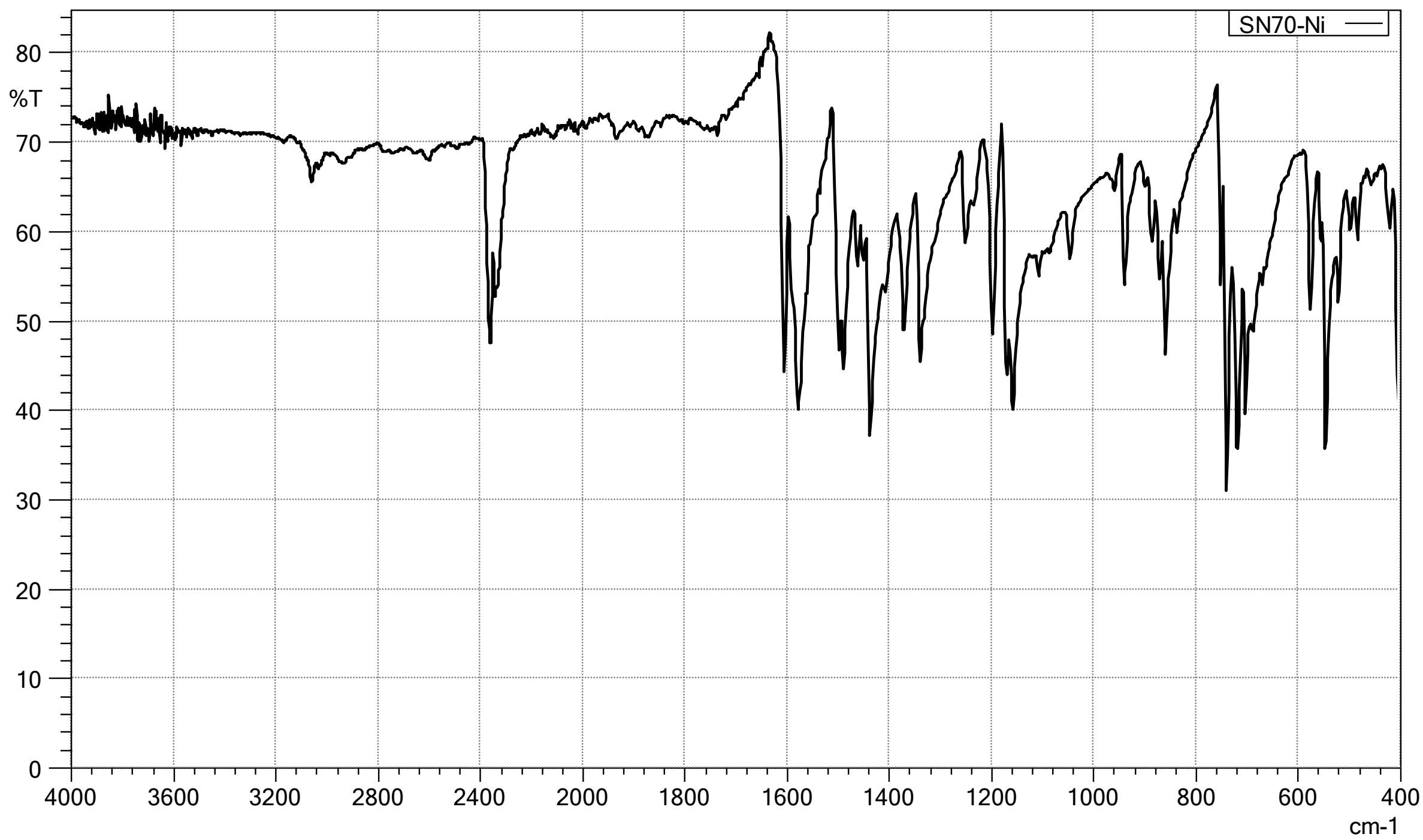

Figure S12. FT-IR spectrum of NiL

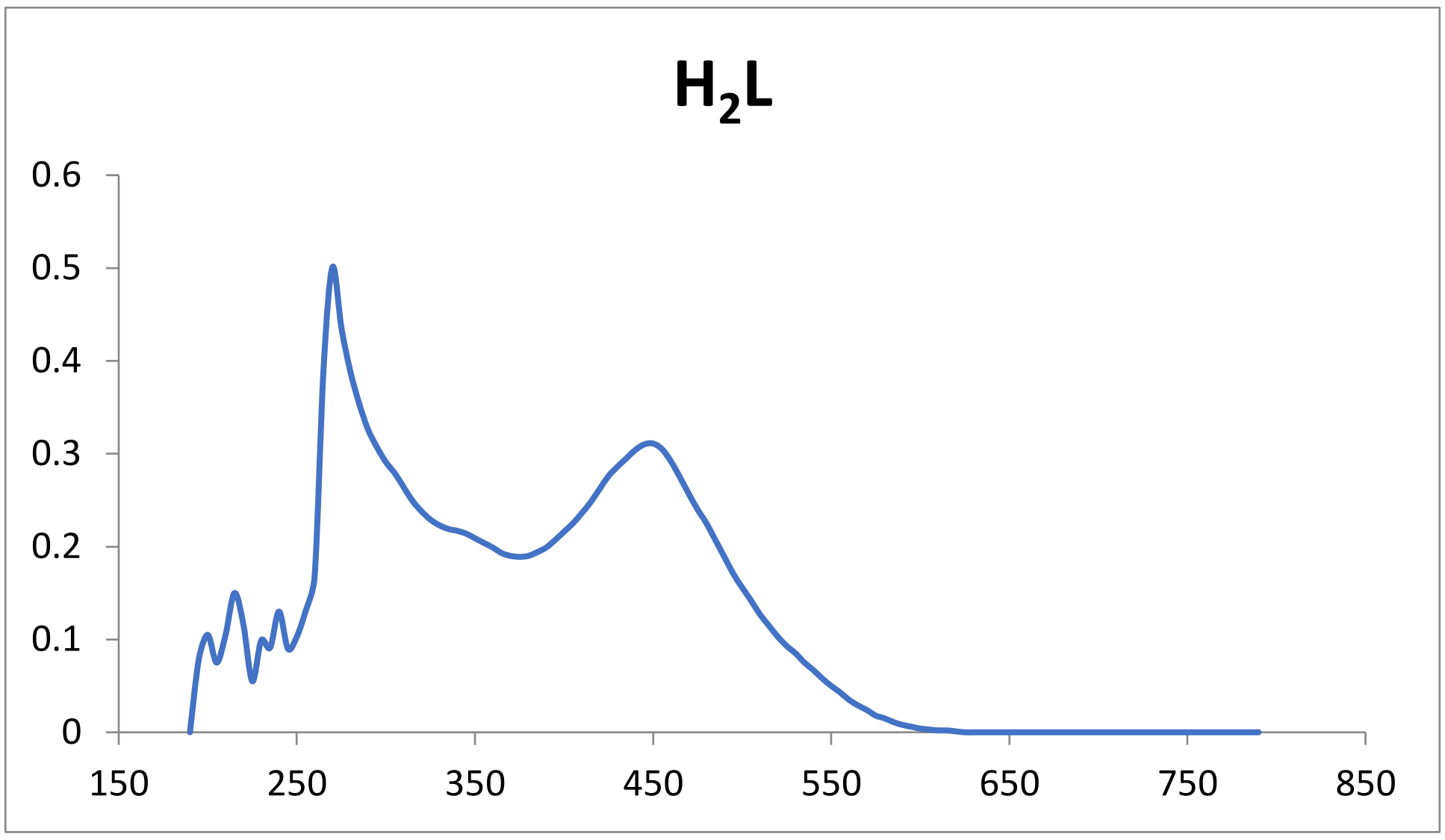

Figure S13. UV-Vis spectrum of H_2L

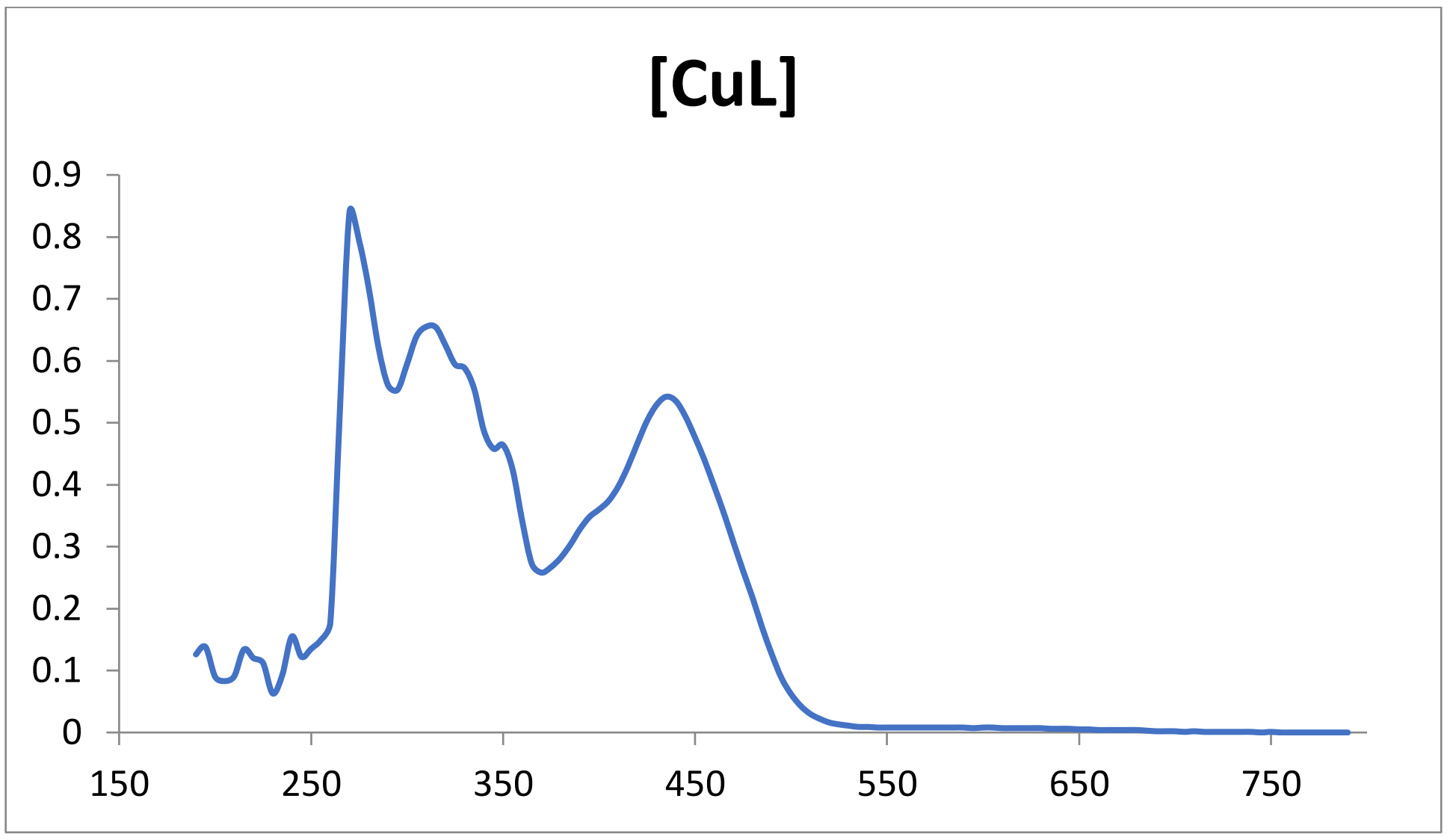

Figure S14. UV-Vis spectrum of CuL

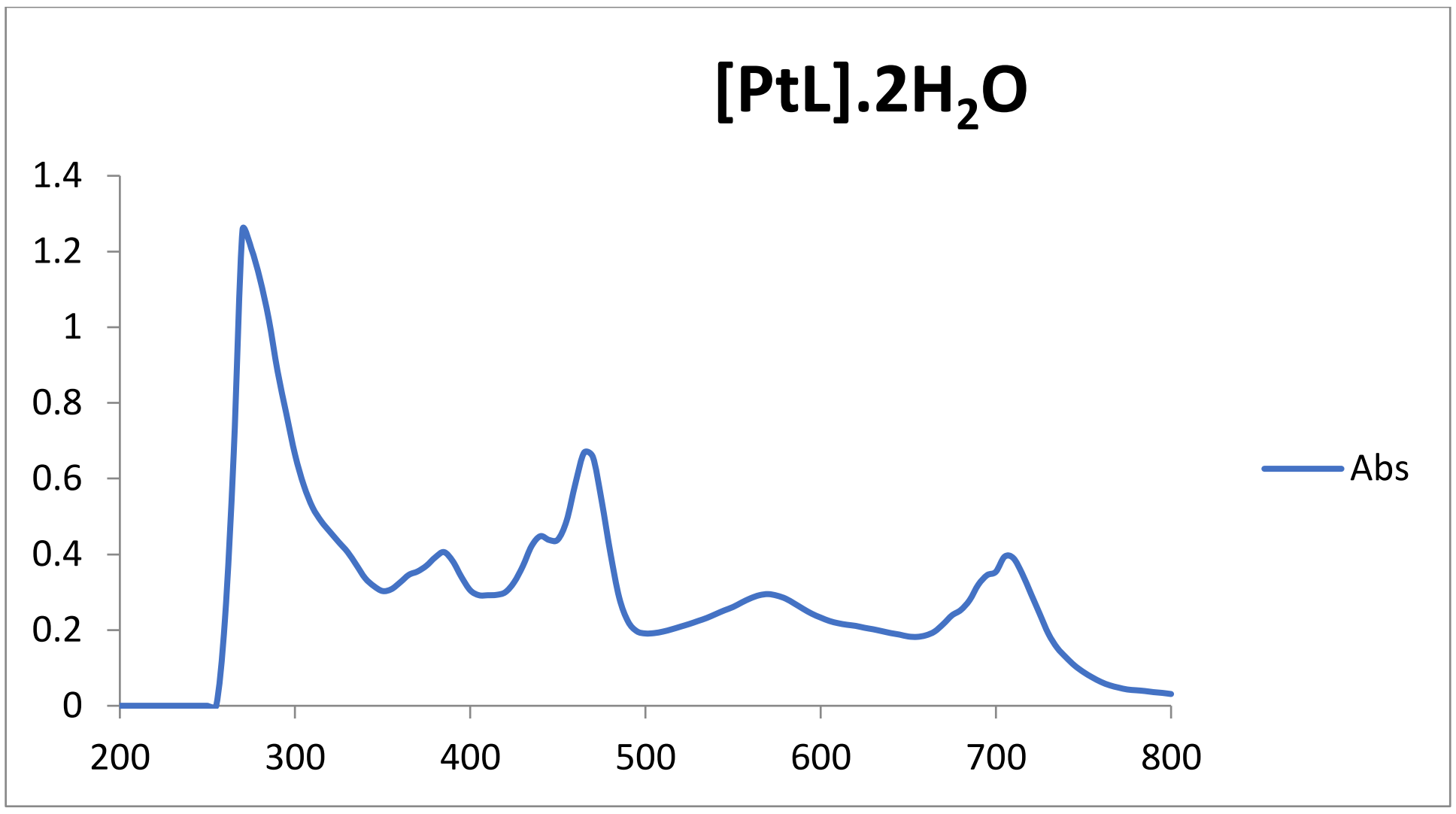

Figure S15. UV-Vis spectrum of PtL

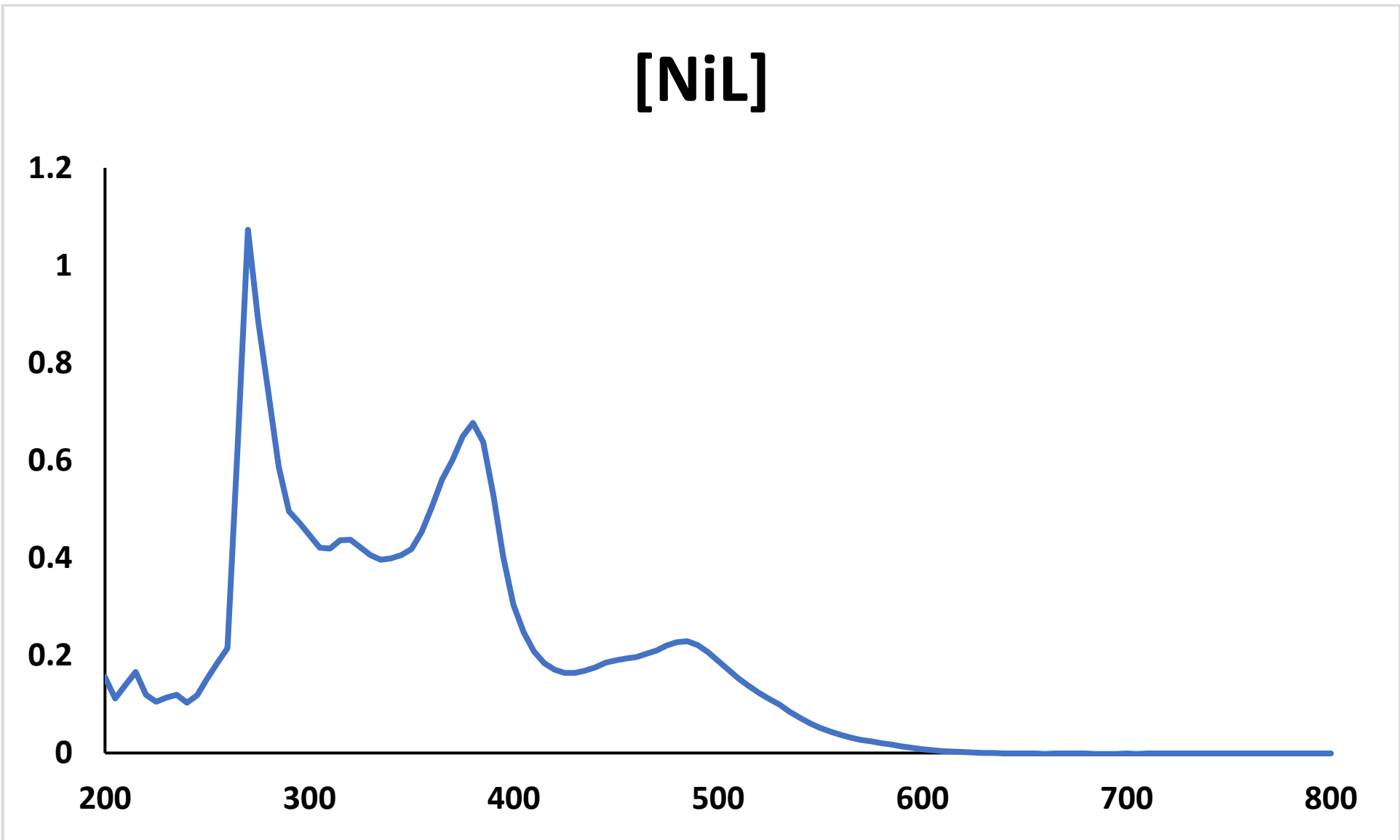

Figure S16. UV-Vis spectrum of NiL

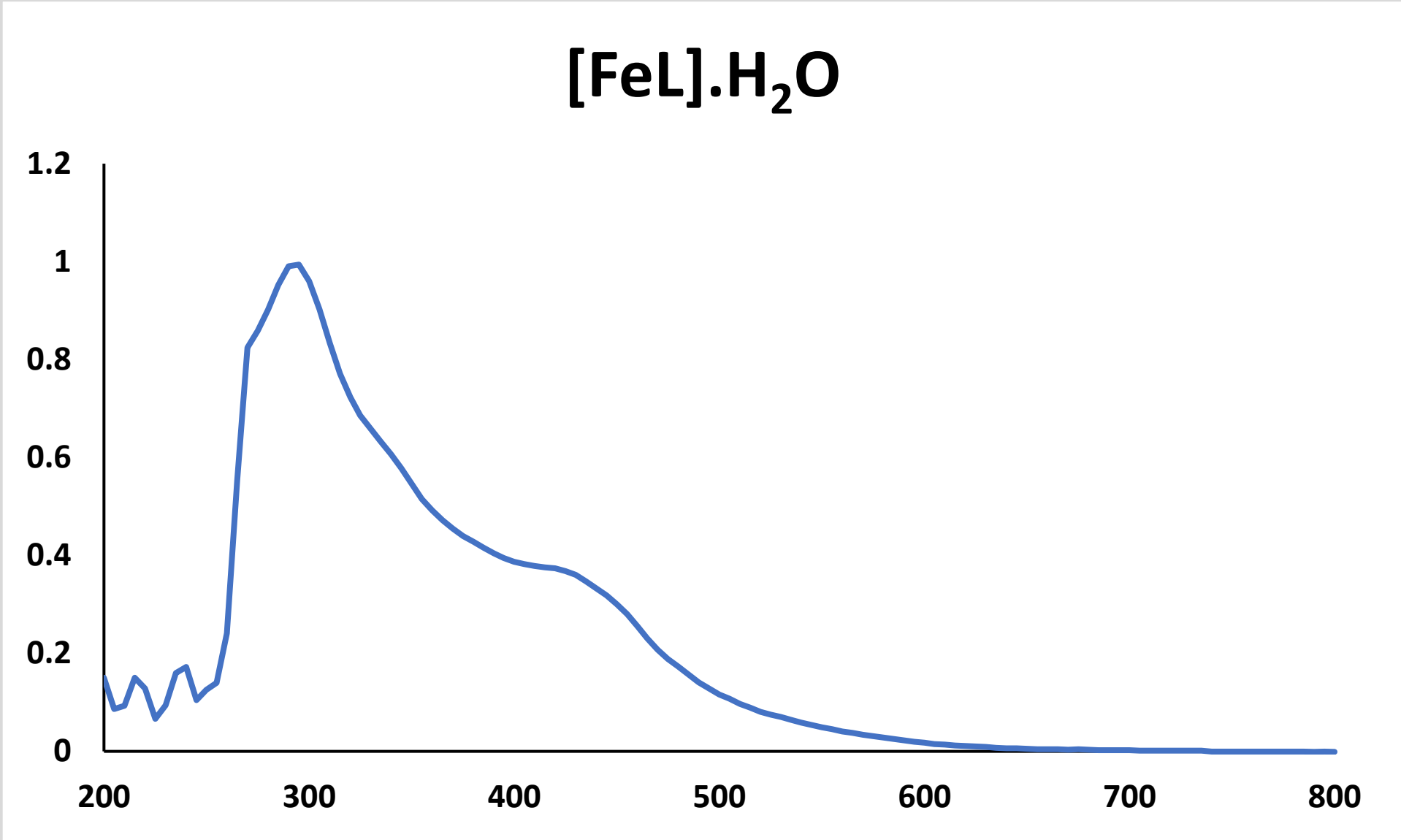

Figure S17. UV-Vis spectrum of FeL

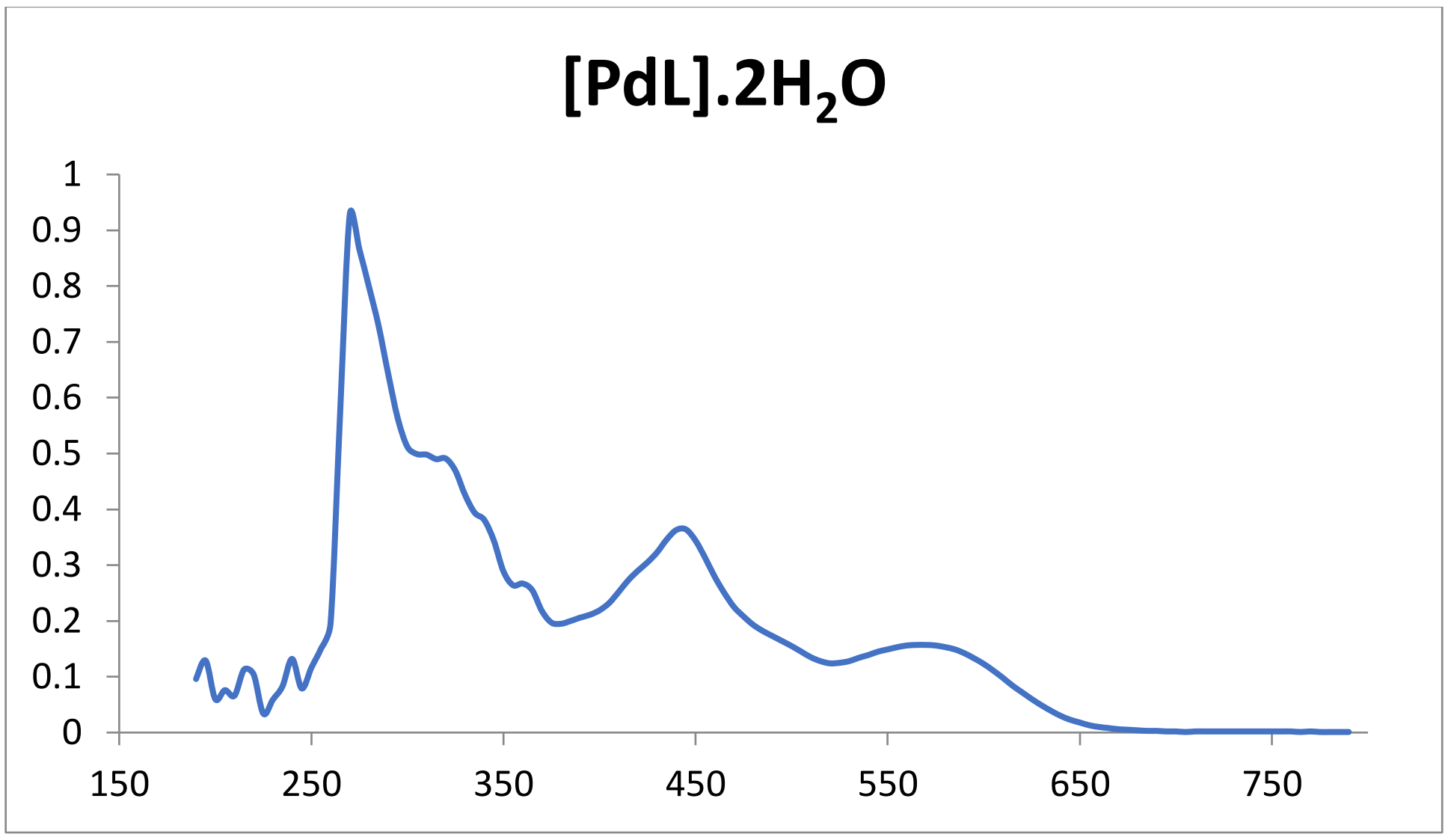

Figure S18. UV-Vis spectrum of PdL

Table S1. The calculated bond lengths (Å) and selected angles (°) of PtL

Bond lengths and angles (Å, °)	B3LYP/LANL2DZ
Bond lenghts (Å)	
Pt1–N1	1.9908
Pt1–N2	1.9908

Pt1–O1	2.0287
Pt1–O2	2.0287
C1-O1	1.3198
C20-O2	1.3198
C7-N1	1.3263
C8-N1	1.4340
C14-N2	1.3263
C13-N2	1.4340
C4-Br1	1.9662
C2-Br2	1.9504
C17-Br3	1.9662
C19-Br4	1.9504
C1-C2	1.4401
C2-C3	1.3881
C3-C4	1.4207
C4-C5	1.3803
C5-C6	1.4355
C6–C7	1.4333
C8-C9	1.4098
C9-C10	1.4019
C10-C11	1.4100
C11–C12	1.4001
C12–C13	1.4098
C13–C8	1.4228
C14-C15	1.4333
C15-C16	1.4355
C16-C17	1.3803
C17-C18	1.4207
C18-C19	1.3881
C19-C20	1.4401
C20-C15	1.4523
Selected bond angles (°)	
N1–Pt1–O2	178.158

N1–Pt1–O1	94.379
N1–Pt1–N2	83.779
N2–Pt1–O1	178.159
N2–Pt1–O2	94.379
O1–Pt1–O2	87.462
Pt1-N1-C7	123.836
Pt1–N1–C8	112.589
Pt1–O1–C1	124.699
Pt1–N2–C14	123.836
Pt1–N2–C13	112.589
Pt1–O2–C20	124.699
Selected torsion angles (°)	
C1-O1-Pt1-N2	-0.009
C1-O1-Pt1-N1	0.009
C1-O1-Pt1-O2	-179.990
C20-O2-Pt1-N1	0.016
C20-O2-Pt1-N2	-0.006
C20-O2-Pt1-O1	-179.993
C6-C7-N1-C8	-179.987
C15-C14-N2-C13	179.995
